# Clinical implications and molecular mechanisms of Cyclin-dependent kinases 4 for patients with hepatocellular carcinoma

**DOI:** 10.1186/s12876-022-02152-w

**Published:** 2022-02-22

**Authors:** Zhong-liu Wei, Xin Zhou, Chen-lu Lan, Hua-sheng Huang, Xi-wen Liao, Shu-tian Mo, Yong-guang Wei, Tao Peng

**Affiliations:** Department of Hepatobiliary Surgery, The First Affiliated Hospital of Guangxi Medical University, Nanning, 530021 Guangxi Zhuang Autonomous Region People’s Republic of China

**Keywords:** Cyclin-dependent kinases, Hepatocellular carcinoma, Prognosis, Biomarker

## Abstract

**Background:**

Hepatocellular carcinoma (HCC) was frequently considered as a kind of malignant tumor with a poor prognosis. Cyclin-dependent kinases (CDK) 4 was considered to be cell-cycle-related CDK gene. In this study, we explored the clinical significance of *CDK4* in HCC patients.

**Methods:**

Data of HCC patients were obtained from The Cancer Genome Atlas database (TCGA) and the Gene Expression Omnibus (GEO) database. Kaplan–Meier analysis and Cox regression model were performed to calculate median survival time (MST) and the hazard ration (HR), respectively. The joint-effect analysis and prognostic risk score model were constructed to demonstrate significance of prognosis-related genes. The differential expression of prognostic genes was further validated using reverse transcription-quantitative PCR (RT-qPCR) of 58 pairs of HCC samples.

**Results:**

*CDK1* and *CDK4* were considered prognostic genes in TCGA and GSE14520 cohort. The result of joint-effect model indicated patients in *CDK1* and *CDK4* low expression groups had a better prognosis in TCGA (adjusted HR = 0.491; adjusted *P* = 0.003) and GSE14520 cohort (adjusted HR = 0.431; adjusted *P* = 0.002). Regarding Kaplan–Meier analysis, high expression of *CDK1* and *CDK4* was related to poor prognosis in both the TCGA (*P* < 0.001 and = 0.001 for *CDK1* and *CDK4*, respectively) and the GSE14520 cohort (*P* = 0.006 and = 0.033 for *CDK1* and *CDK4*, respectively). However, only *CDK4* (*P* = 0.042) was validated in RT-qPCR experiment, while *CDK1* (*P* = 0.075) was not.

**Conclusion:**

HCC patients with high *CDK4* expression have poor prognosis, and *CDK4* could be a potential candidate diagnostic biomarker for HCC.

**Supplementary Information:**

The online version contains supplementary material available at 10.1186/s12876-022-02152-w.

## Background

In 2020, liver cancer was known to rank sixth among diagnosed malignant cancers worldwide and the third leading cause of cancer death, ranking second in terms of cancer death for males [1]. 75–85% of primary liver cancer was HCC [1]. The main risk factors were chronic hepatitis B or C, aflatoxin B1 exposure, excessive alcohol intake and alcohol-related liver disease [1, 2]. Metabolic diseases such as type 2 diabetes (T2DM) and non-alcoholic fatty liver (NAFLD) are high-risk factors for HCC [3, 4]. NAFLD can progress to nonalcoholic steatohepatitis and then to HCC, and T2DM increases the risk of HCC by a factor of 3 through the PTEN/P13K/Akt and MAPK kinase molecular pathway [3–5]. Although there were many well-established diagnoses for HCC, including computed tomography, ultrasonography, serum tumor markers and magnetic resonance imaging [2], HCC patients are usually in advanced liver failure when they develop symptoms and are usually untreatable [6]. Therefore, patients can be diagnosed early to obtain longer overall survival and it is necessary to explore molecular biomarkers to provide early diagnosis and prognostic assessment for HCC patients.

The CDKs genes family play a vital role in cell division and modulating transcription [7]. A total of 21 genes in the CDKs gene family were divided into 11 subfamilies, of which CDK1 (*CDK1*, *CDK2*, *CDK3*), CDK4 (*CDK4*, *CDK6*) subfamily were considered to be cell-cycle-related subfamilies [7, 8]. Cell cycle regulators were frequently mutated in the tumor, including overexpression of *CDKs* [9]. There are many studies reporting the involvement of *CDK1* and *CDK4* subfamilies in the progression of multiple cancer [10–20]. However, the relationship between *CDK1-4, 6* expression and the risk of HCC patients was rarely reported. Thus, this study aims to explore the role of *CDK1-4, 6* expressions in HCC patients based on public cancer data.

## Materials and methods

### Data source

The mRNA expression and clinical information of HCC in the TCGA database were obtained from the University of California, Santa Cruz Xena (UCSC Xena, https://xenabrowser.net/datapages/) [21]. The GSE14520 dataset from the GEO database was analyzed. The platform of GSE14520 was GPL3921, which collected the mRNA expression levels of 225 HCC tissues and 220 matched liver tissues. The clinical information and mRNA gene expression matrix of HCC were downloaded from GSE14520 on the GEO website (https://www.ncbi.nlm.nih.gov/geo) [22, 23]. The limma package was used to process and normalize the raw data of the GSE14520 gene expression matrix in R platform. The gene expression and clinical data from Chinese HCC (CHCC) patients were accessed via the National Omics Data Encyclopedia (NODE) website (https://www.biosino.org/node/project/detail/OEP000321) [24].

### Tissues processing and RT-qPCR experiments

In RT-qPCR experiment, a total of 58 pairs of tumor and adjacent normal liver tissues (> 3 cm margin from the tumor) of patients pathologically diagnosed as HCC in The First Affiliated Hospital of Guangxi Medical University were collected for further analysis. Inclusion criteria: Patients received no other non-surgical treatments before surgery and hospitalization time was from December 2016 to July 2018. HCC patients with follow-up time < 3 months were excluded. Small pieces of HCC and adjacent normal liver tissues were stored in RNAstore Reagent (Tiangen Biotech Co., Ltd.) at -80℃. According to the manufacturer’s instructions, RNA was extracted from tissues by the Trizol method and reversed into cDNA via PrimeScriptTMRT reagent kit (Takara Bio, Inc.). Primers GAPDH, CDK1, and CDK4 were purchased from TsingKe Biotech Co., Ltd. and their sequences (5'-3') were as follows: GAPDH, forward GTCAGCCGCATCTTCTTT, reverse CGCCCAATACGACCAAAT; CDK1, forward TTTCTTTCGCGCTCTAGCCA, reverse GGTAGATCCGCGCTAAAGGG; CDK4, forward AGCCAGAGAACATTCTGGTGACA, reverse TCGGCTTCAGAGTTTCCACAG. DEPC-treated water and FastStart Universal SYBR Green Master (ROX) were purchased from Sangon Biotech Co., Ltd., and Roche Diagnostics (Shanghai) Co., Ltd., respectively. The reaction cycle of PCR is as follows: hold the stage at 95 degrees for 35 s, then 40 cycles of PRC stage at 95 degrees for 5 s and 55 degrees for 34 s, and finally melt curve stage at 95 degrees for 15 s, 60 degrees for 1 min and 95 degrees for 15 s. The relative expression of mRNAs in tissues was calculated by the 2-∆∆Ct method.

### Ethical approval

This study was approved by the Ethical Review Committee of the First Affiliated Hospital of Guangxi Medical University and informed consent of all HCC patients participating in this study was provided.

### Bioinformatics analysis and correlation analysis

The Gene Ontology (GO) analysis and Kyoto Encyclopedia of Genes and Genomes (KEGG) pathway analysis were used to study the potential biological functions and potential metabolic pathways of *CDK1-4,6* by the clusterProfiler package in the R software [25]. The interactive gene‑gene networks and protein–protein interaction (PPI) networks were depicted by the GeneMANIA tool in the Cytoscape software v.3.6.1 [26, 27] and STRING (https://string-db.org) [28], respectively. Pearson’s correlation matrix was depicted correlations among *CDK1-4, 6* genes by the ggcorrplot package in the R software.

### Diagnostic values assessment and survival analysis

Receiver operating characteristic (ROC) curve was completed by GraphPad Prism v.8 to investigate the diagnostic value of *CDK1-4, 6* genes with differential expression for HCC. Univariate Cox regression model was performed to identify the relationship between clinical information and prognosis of HCC patients. Clinical information statistically associated with prognosis in the univariate Cox regression model (*P* < 0.05) was selected as the adjusted factors for the multivariate Cox regression model. According to the median expression levels of *CDK1-4, 6* of tumor tissues, patients were classified into high and low expression groups. The multivariate cox regression model and Kaplan‑Meier survival analysis were performed to explore the relationship between *CDK1-4, 6* gene expression and overall survival (OS) in HCC patients. Only *CDK1* and *CDK4* were found to be statistically associated with OS in the multivariate cox regression model. Therefore, *CDK1* and *CDK4* were selected as prognosis-related *CDK* genes in HCC patients. Joint-effect analysis was performed to evaluate the combined effect of *CDK1* and *CDK4*. In the TIMER2.0 (http://timer.comp-genomics.org/) [29] and Gene Expression Profiling Interactive Analysis (GEPIA, http://gepia.cancer-pku.cn/) [30] website, we queried the expression of *CDK1* and *CDK4* genes in different cancers and the relationship between the expression of *CDK1* and *CDK4* genes and *TP53* gene mutation, a common mutation site in liver cancer. The prognostic value of *CDK1* and *CDK4* genes in liver cancers were obtained from the Kaplan–Meier Plotter website (https://kmplot.com/analysis/) [31].

### Prognostic signature construction

To further explore the influence of *CDK1* and *CDK4* expression levels on the prognosis of HCC patients. The prognostic risk score model was established by including each the prognosis-related genes respectively weighted by their regression coefficients (β) from the multivariate Cox regression model. To assess the predictive value of the model, the area under the curve (AUC) of the time-dependent ROC curve was completed by the survivalROC package to indicate the predictive accuracy of the model for 1-, 2-, 3- and 5-year survival.

### Gene set enrichment analysis

The gene set enrichment analysis (GSEA) was used to explore potential biological mechanisms that prognosis-related *CDK* genes may be involved in. [32, 33] The referenced gene sets derived from Molecular Signatures Database (MSigDB) of c2 (c2.all.v7.0. symbols) and c5 (c5.all.v7.0.symbols) [34]. C2 gene set contained two subsets of Chemical and genetic perturbations and Canonical pathways and c5 gene set derived from GO annotations [34]. The statistically significant results satisfied the following criteria: *P* < 0.05 and false discovery rate < 0.25.

### Statistical analysis

T-test was used to assess differential expression of *CDK1-4, 6* genes between HCC and matched normal tissues. Correlation among *CDK1-4, 6* genes was assessed by Pearson’s correlation coefficients. MST was obtained by Kaplan–Meier survival analysis with the log-rank test. Association of *CDK* gene expression levels and clinical information with OS was assessed by HR and 95% confidence interval (CI) was calculated by Cox regression model. All statistical analyses were done by SPSS v.22.0 software (IBM Corporation, USA). Kaplan–Meier survival curve, scatter plots and ROC curve was depicted by GraphPad Prism v.8 software. Scatter plots, heat maps, histograms and matrix plots were depicted by the R platform (version 3.6.3). *P* < 0.05 was considered to be statistically significant in this study.

## Results

**Fig. 1:**
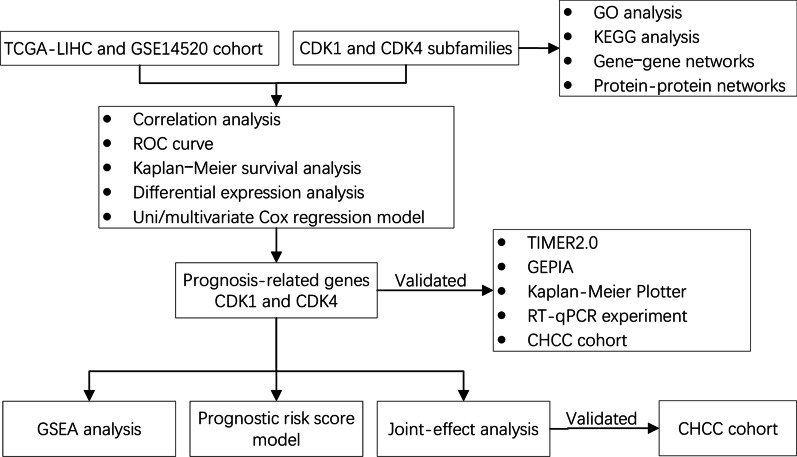
Schematic diagram of the study design

The design of this study is displayed in the flow chart (Fig. 1).

### Data source

TCGA cohort included 370 HCC tissues and 50 adjacent normal liver tissues and corresponding 370 patients’ prognostic information. In GSE14520 dataset, the majority of patients were Hepatitis B virus (HBV)-infected patients. To reduce confounding factors, 212 HCC tissues and 204 matched normal liver tissues from 212 HBV-infected HCC patients and the corresponding clinical information were retained in the GSE14520 cohort. In the CHCC cohort, gene expression was acquired in tumor and normal liver tissues from 159 Chinese HCC patients who underwent radical resection.

### Bioinformatics analysis and correlation analysis

**Fig. 2:**
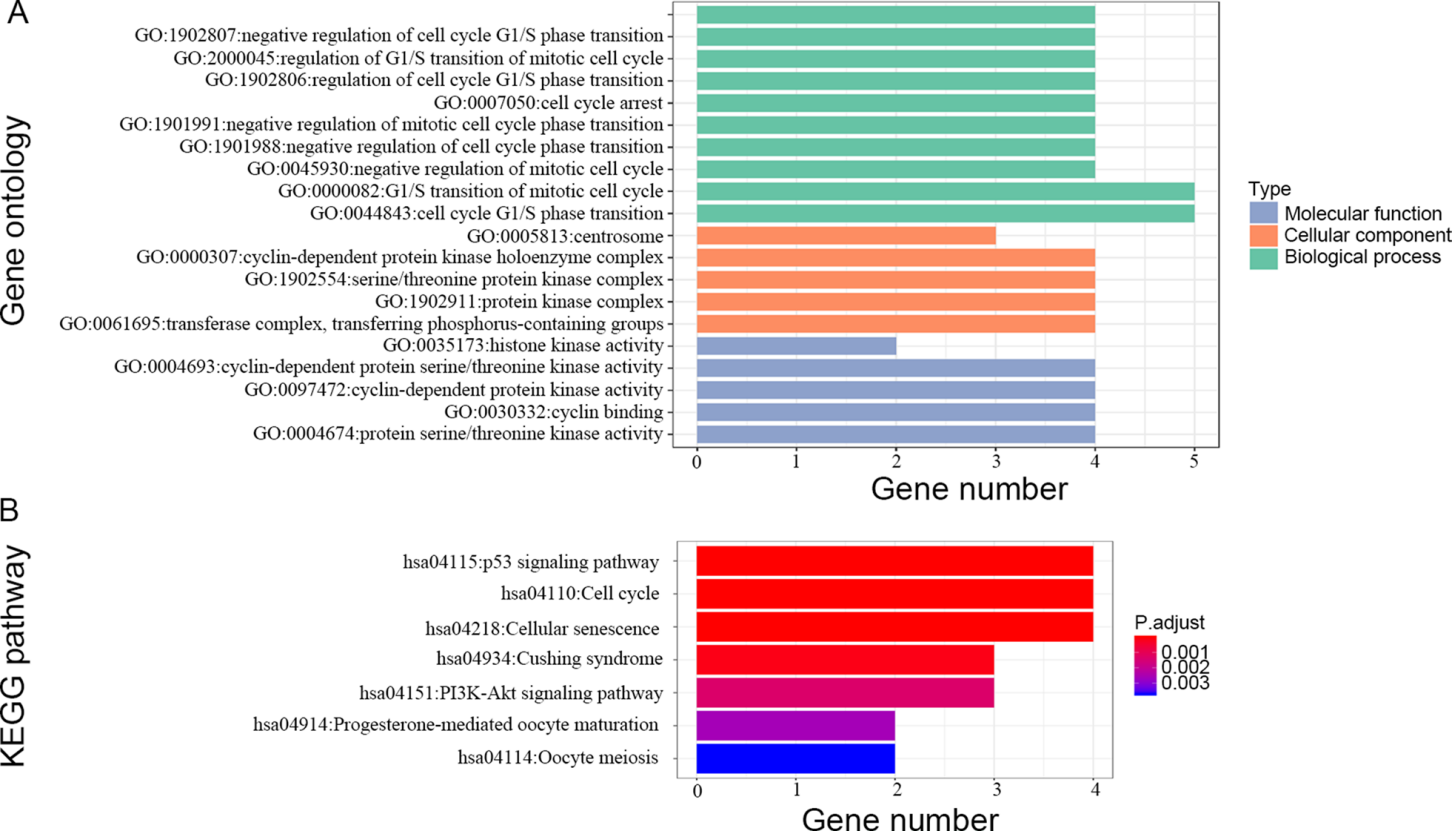
GO enrichment and KEGG pathway analysis of *CDK1-4, 6*. GO analysis (**A**); KEGG pathway analysis (**B**)

**Fig. 3:**
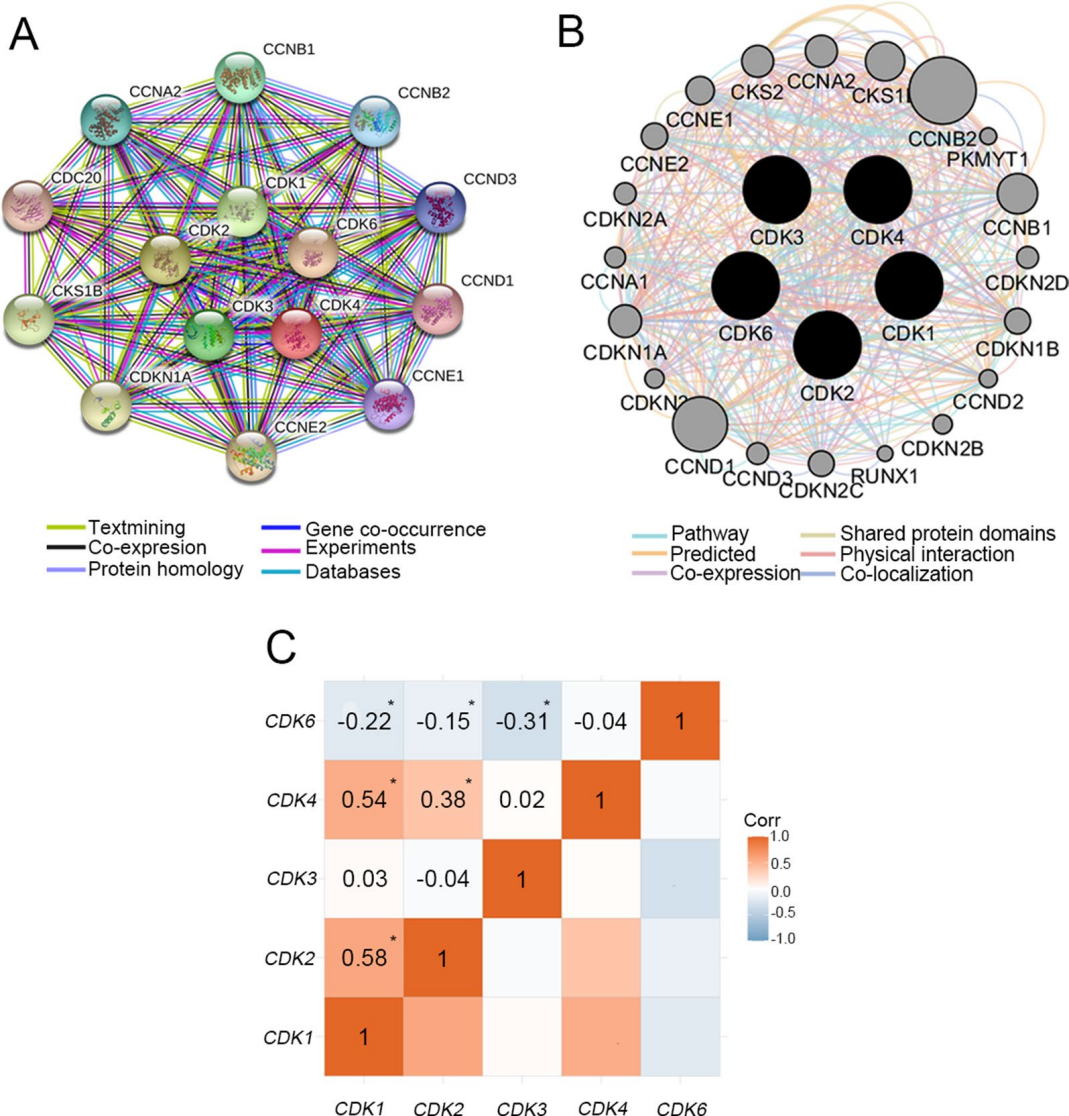
The protein–protein interaction networks among *CDK1-4, 6* proteins with other proteins (**A**) and gene–gene interaction networks among *CDK1-4, 6* genes with other genes (**B**). Matrix graphs of Pearson's correlations cofficient of *CDK1-4, 6* gene expressions in the TCGA database (**C**). *Note*: *P < 0.05

The results of GO analysis suggested that biological functions (Cellular component, Biological process, Molecular function) of *CDK1-4, 6* were involved in regulation of cell cycle, serine/threonine protein kinase complex and protein serine/threonine kinase activity, etc. (Fig. 2A) The KEGG pathway analysis indicated pathways involved in *CDK1-4, 6* were enriched cell cycle, p53 signaling pathway, cellular senescence and PI3K-Akt signaling pathway (Fig. 2B and Additional files 1, 2: Figure S1-2) [35–37]. The PPI networks suggested that CDK1-4, 6 proteins were associated with Cyclins (CCN) family proteins, CDC20, CDKN1A and CKS1B (Fig. 3A). Moreover, the gene-gene interaction network showed that nine CCN family number genes (*CCNA1*, *CCNA2*, *CCNB1*, *CCNB2*, *CCND1*, *CCND2*, *CCND3*, *CCNE1* and *CCNE2*) and other genes (*CDKN2A*, *RUNX1*, CDKN1A, and so on) also were associated with *CDK1-4, 6* (Fig. 3B). Pearson's correlation coefficient of *CDK1-4, 6* was used to manifest the correlation among genes (Fig. 3C). The results suggested that *CDK1-4, 6* genes had a certain correlation with each other in TCGA.

### Diagnostic values assessment

**Fig. 4:**
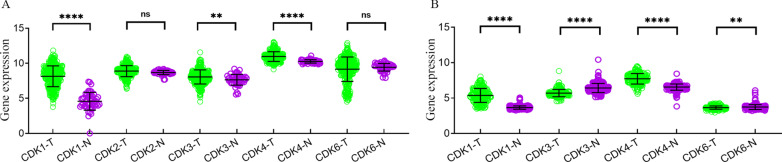
Scatter plot of expression level of *CDK1-4, 6* genes between tumor tissue and adjacent normal liver tissues in TCGA cohort (**A**) and in GSE14520 cohort (**B**). *Note*: *CDK2* was unavailable in GSE14520 of GEO database. NS: p > 0.05; *p < 0.05; **p < 0.01; ***p < 0.001; ****p < 0.0001

**Fig. 5:**
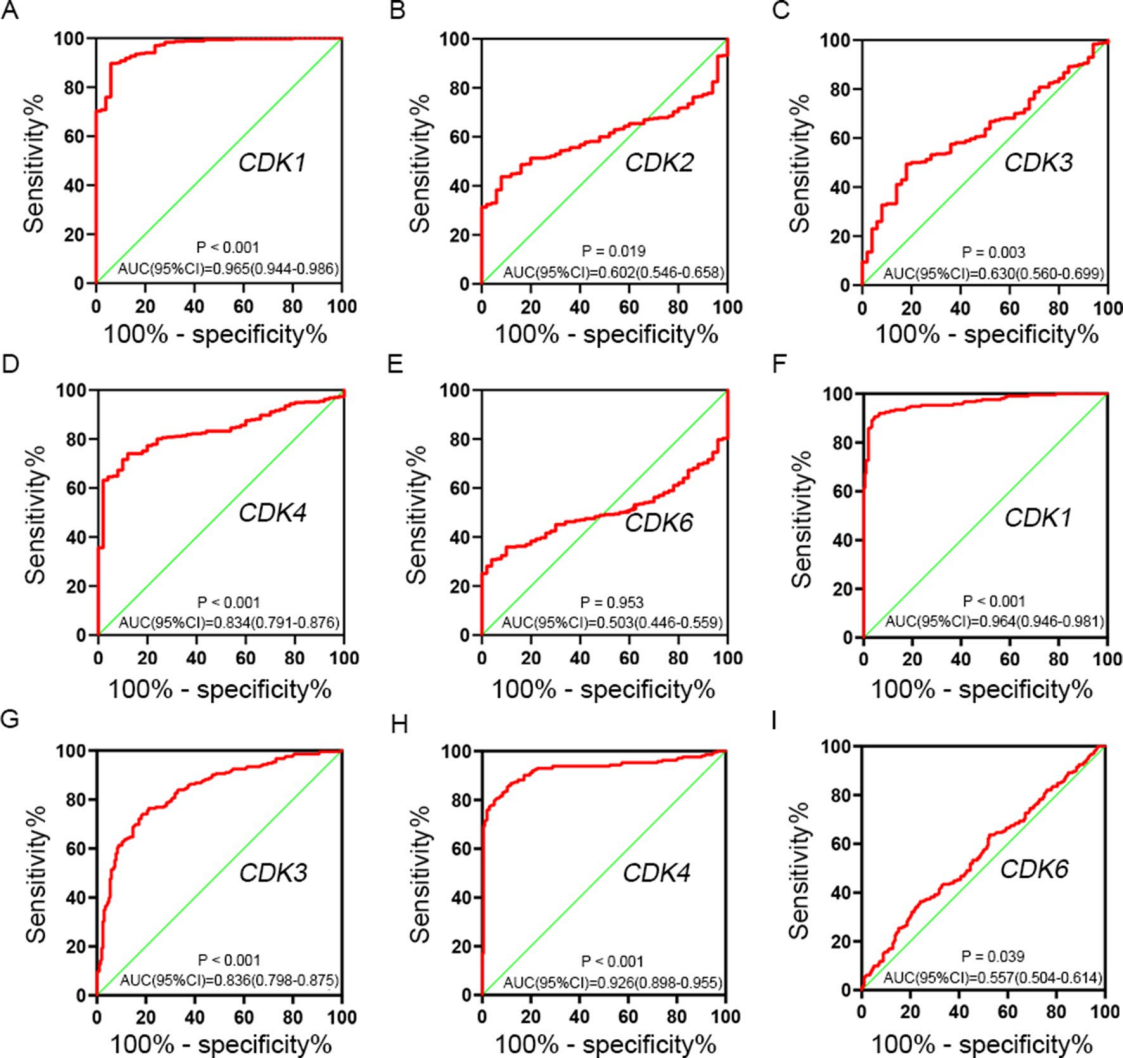
The ROC curves of *CDK* gens in distinguish HCC tumor tissue and adjacent normal tissues. The ROC curves of *CDK1* (**A**), *CDK2* (**B**), *CDK3* (**C**), *CDK4* (**D**), *CDK6* (**E**) in TCGA cohort; the ROC curves of *CDK1* (**F**), *CDK3* (**G**), *CDK4* (**H**), *CDK6* (**I**) in GSE14520 cohort. *Note*: *CDK2* was unavailable in GSE14520 of GEO database

The expression of *CDK1-4, 6* genes in different tissues was shown in the scatter plot (Fig. 4), and a total of 3 *CDK* genes (*CDK1, CDK3* and *CDK4*) were found to be overexpressed in HCC tissues of TCGA cohort. Similarly, *CDK1*, *CDK3*, *CDK4* and *CDK6* were found to be overexpressed in HCC tissues of GSE14520 cohort. To evaluate the diagnostic value of *CDK1-4, 6* genes expression, the ROC curve indicated that *CDK1* (*P* < 0.001 and AUC = 0.965) and *CDK4* (*P* < 0.001 and AUC = 0.834) had potential predictive value in TCGA cohort (Fig. 5A–E). Moreover, a total of 3 genes in GSE14520 cohort, *CDK1* (*P* < 0.001 and AUC = 0.964), *CDK3* (*P* < 0.001 and AUC = 0.836) and *CDK4* (*P* < 0.001 and AUC = 0.926) suggested potential diagnostic value (Fig. 5F–I). In particular, *CDK1* and *CDK4* showed high accuracy in both TCGA and GSE14520 cohort (*P* < 0.001 and AUC > 0.800).

### Survival analysis

**Fig. 6:**
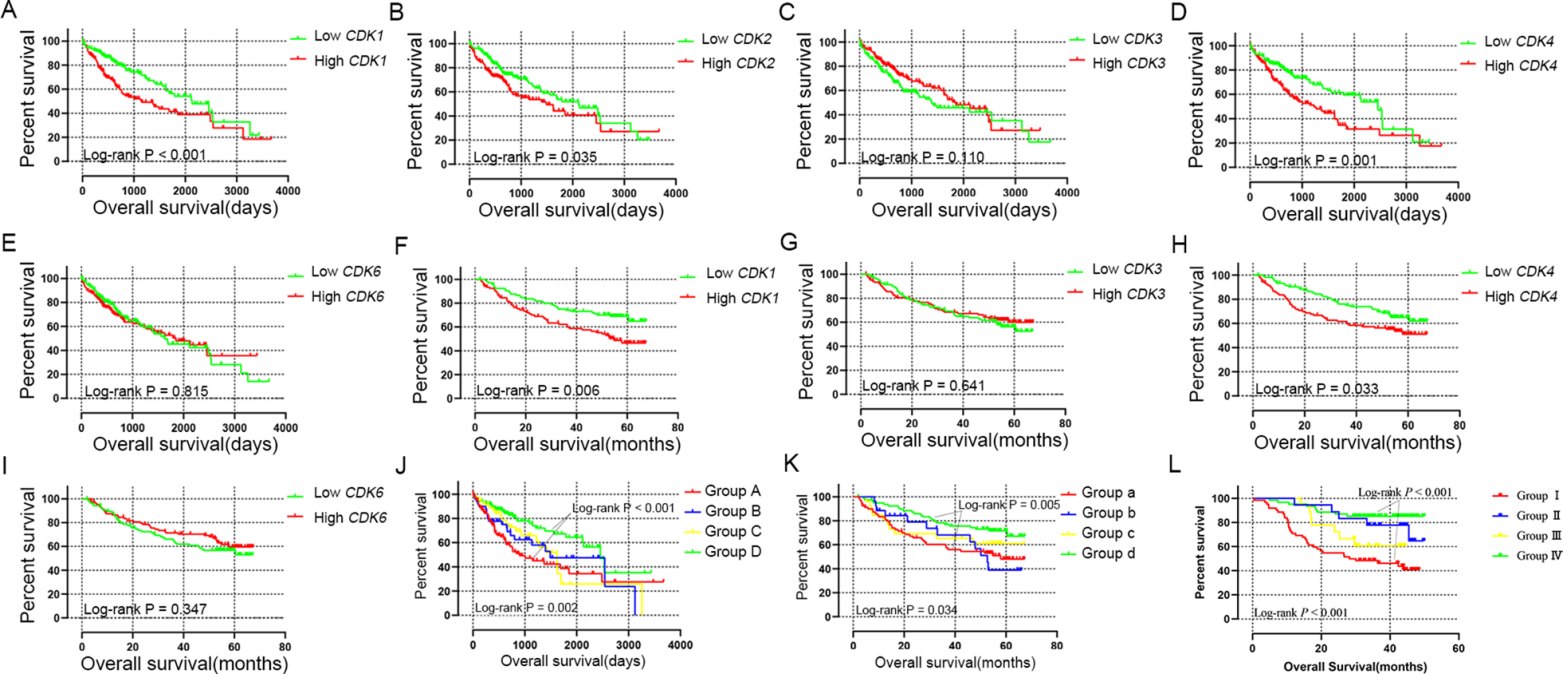
The Kaplan–Meier survival curves for *CDK* gens in HCC. Overall survival curves were plotted for *CDK*1 (**A**), *CDK2* (**B**), *CDK3* (**C**), *CDK4* (**D**) and *CDK6 * (**E**) in TCGA cohort*;* Overall survival curves were plotted for *CDK1* (**F**), *CDK3* (**G**), *CDK4* (**H**) and *CDK6* (**I**) in GSE14520 cohort. The Kaplan–Meier survival curves for joint-effects analysis of *CDK*1 and *CDK4* in HCC of TCGA cohort (**J**), GSE14520 cohort (**K**) and CHCC cohort (**L**). *Note*: *CDK2* was unavailable in GSE14520 of GEO database

Kaplan‑Meier survival analysis (Fig. 6A–I) indicated that high expression of *CDK1* and *CD*K4 had statistically significant worse prognosis in both TCGA cohort (Fig. 6A, D; *P* < 0.001 and = 0.001 for *CDK1* and *CDK4*, respectively) and GSE14520 cohort (Fig. 6F, H; *P* = 0.006 and = 0.033 for *CDK1* and *CDK4*, respectively).

The clinical information for the TCGA cohort of 370 HCC was presented in Table 1. Radical resection (*P* = 0.007) and III or IV TNM stage (*P* < 0.001) were statistically significant for OS. The clinical information for GSE14520 cohort of 212 HBV-infected HCC patients was presented in Table 2. Tumor size (*P* = 0.002), cirrhosis (*P* = 0.041), BCLC stage (*P* = 0.050, 0.004 and < 0.001 for A stage, B stage and C stage, respectively), serum AFP (*P* = 0.049) and TNM stage (*P* = 0.005 and < 0.001 for II stage and III or IV stage, respectively) were statistically significant for OS in HCC patients. Tumor size (*P* < 0.001), tumor thrombus (*P* = 0.005), preoperative AFP (*P* < 0.001) and BCLC stage (*P* = 0.014 and < 0.001 for B stage and C stage, respectively) were statistically associated with OS in the CHCC cohort (Additional file 10: Table S1). In TCGA, GSE14520 and CHCC cohort, the above clinical information statistically relevant to OS was considered as prognostic-related information for adjustion in the multivariate Cox regression model.

**Table 1:** Clinical data of 370 HCC patients from TCGA database

Variables	Patient (n = 370)	OS			
		No. of events	MST (days)	HR (95% CI)	P
Age (years)					
≤ 60	177	55	2532	1	
> 60	193	75	1622	1.246 (0.879–1.766)	0.217
Gender					
Female	121	51	1490	1	
Male	249	79	2486	0.817 (0.573–1.164)	0.262
Race					
Asian	157	44	NA	1	
White + other	203	81	1386	1.309 (0.904–1.896)	0.154
Alcohol consumption^a^					
No	234	84	1694	1	
Yes	117	40	1624	1.026 (0.703–1.496)	0.896
Ishak fibrosis score^b^					
0—No Fibrosis	74	30	2131	1	
1,2—Portal Fibrosis	31	9	1372	0.917 (0.429–1.962)	0.823
3,4—Fibrous Speta	28	6	NA	0.682 (0.281–1.654)	0.397
5—Nodular Formation and Incomplete Cirrhosis	9	2	1386	0.750 (0.177–3.167)	0.695
6—Established Cirrhosis	69	17	NA	0.766 (0.418–1.403)	0.388
TNM stage^c^					
I	171	42	2532	1	
II	85	26	1852	1.427 (0.874–2.330)	0.155
III or IV	90	48	770	2.764 (1.823–4.190)	< 0.001
Histologic Grade^d^					
G1	55	18	2116	1	
G2	177	60	1685	1.181 (0.697–2.000)	0.537
G3	121	43	1622	1.233 (0.711–2.140)	0.456
G4	12	5	NA	1.693 (0.626–4.584)	0.300
Serum AFP^e^					
≤ 400 ng/ml	213	62	2456	1	
> 400 ng/ml	64	22	2486	1.055 (0.645–1.724)	0.832
Radical resection^f^					
R0	323	110	1852	1	
R1/R2/RX	40	17	837	2.030 (1.213–3.395)	0.007
Micro vascular invasion^g^					
No	206	60	2131	1	
Yes	108	36	2486	1.351 (0.892–2.047)	0.155
Child–Pugh score^h^					
A	216	59	2542	1	
B/C	22	9	1005	1.614 (0.796–3.270)	0.184

OS, overall survival; MST, median survival time; HR, hazard ratio; CI, confidence interval; AFP, α-fetoprotein; NA, not available

^a^Alcohol consumption information is not available for 19 patients

^b^Ishak fibrosis score information is not available for 159 patients

^c^umor stage information is not available for 24 patients

^d^Histologic grade information is not available for 5 patients

^e^Serum AFP information is not available for 93 patients

^f^Radical resection information is not available for 7 patients

^g^Micro vascular invasion information is not available for 56 patients

^h^Child-Pugh score information is not available for 132 patients

**Table 2:** Clinical data of 212 HBV-related HCC patients in the GSE14520 of GEO database

Variables	Patient (n = 212)	OS			
		No. of events	MST (months)	HR (95% CI)	P
Age (years)					
≤ 60	175	69	NA	1	
> 60	37	13	NA	0.864 (0.478–1.564)	0.63
Gender					
Female	29	8	NA	1	
Male	183	74	NA	1.704 (0.821–3.534)	0.152
Multinodular					
Single	167	59	NA	1	
Multiple	45	23	47.9	1.607 (0.992–2.604)	0.054
Tumor size^a^					
≤ 5 cm	137	46	NA	1	
> 5 cm	74	36	53.3	1.975 (1.274–3.060)	0.002
Cirrhosis					
No	17	2	NA	1	
Yes	195	80	NA	4.335 (1.065–17.638)	0.041
BCLC stage					
0	20	2	NA	1	
A	143	48	NA	4.119 (1.001–16.951)	0.050
B	22	12	46.1	8.992 (2.005–40.320)	0.004
C	27	20	13.6	18.993 (4.419–81.632)	< 0.001
Serum AFP^b^					
≤ 300 ng/ml	115	39	NA	1	
> 300 ng/ml	94	43	NA	1.546 (1.002–2.385)	0.049
ALT					
< 50U/L	124	46	NA	1	
≥ 50U/L	88	36	NA	1.095 (0.708–1.693)	0.684
TNM stage					
I	89	20	NA	1	
II	76	32	NA	2.214 (1.265–3.873)	0.005
III or IV	47	30	18	5.197 (2.930–9.218)	< 0.001

HBV, hepatitis B virus; BCLC, Barcelona Clinic Liver Cancer; AFP, α-fetoprotein; MST, median survival time; OS, overall survival; HR, hazard ratio; CI, confidence interval; NA, not available

^a^Information of tumor size was unavailable in 1 patients

^b^Information of serum AFP was unavailable in 3 patients

After adjusting tumor stage and radical resection, the multivariate Cox regression model of TCGA cohort (Table 3) suggested that high expression of *CDK1* (adjusted HR = 1.541; adjusted *P* = 0.028) and *CDK4* (adjusted HR = 1.721; adjusted *P* = 0.005) were statistically related to OS. In GSE14520 cohort (Table 4), tumor size, cirrhosis, and BCLC stage were considered as adjusted factors in the multivariate Cox regression model, which suggested that high expression of *CDK1* (adjusted HR = 2.237; adjusted *P* < 0.001) and *CDK4* (adjusted HR = 1.579; adjusted *P* = 0.044) were statistically related to OS in HBV-infected HCC patients.

**Table 3:** Relationship between CDK gene expression and HCC prognosis in TCGA cohort

Variables	Patient (n = 370)	OS					
		No. of events	MST (days)	HR (95% CI)	P	Adjusted HR (95% CI)	Adjusted P^a^
CDK1							
LOW	185	53	2131	1		1	
HIGH	185	77	1149	1.796 (1.265–2.551)	0.001	1.541 (1.048–2.266)	0.028
CDK2							
LOW	185	61	2116	1		1	
HIGH	185	69	1490	1.450 (1.025–2.051)	0.036	1.305 (0.896–1.900)	0.165
CDK3							
LOW	185	65	13,786	1		1	
HIGH	185	65	1852	0.775 (0.534–1.067)	0.111	0.934 (0.640–1.363)	0.723
CK4							
LOW	185	54	2456	1		1	
HIGH	185	76	1229	1.779 (1.253–2.526)	0.001	1.721 (1.179–2.513)	0.005
CDK6							
LOW	185	66	1624	1		1	
HIGH	185	64	1852	1.042 (0.738–1.471)	0.815	1.068 (0.733–1.554)	0.733

OS, overall survival; MST, median survival time; HR, hazard ratio; CI, confidence interval

^a^Adjusted for tumor stage and radical resection

**Table 4:** Relationship between CDK gene expression and HCC prognosis in GSE14520 cohort

Expression	Patient (n = 212)	OS					
		No. of events	MST (months)	HR (95% CI)	P	Adjusted HR ^a^ (95% CI)	Adjusted P^a^
CDK1							
Low	106	33	NA	1		1	
High	106	49	54.8	1.848 (1.187–2.876)	0.07	2.237 (1.424–3.514)	< 0.001
CDK3							
Low	106	43	NA	1		1	
High	106	39	NA	0.902 (0.585–1.392)	0.642	1.116 (0.716–1.742)	0.628
CDK4							
Low	106	35	NA	1		1	
High	106	47	NA	1.605 (1.036–2.487)		1.579 (1.013–2.461)	0.044
CDK6							
Low	106	44	NA	1		1	
High	106	38	NA	0.812 (0.526–1.254)	0.348	0.88 (0.567–1.365)	0.568

HBV, hepatitis B virus; MST, median survival time; OS, overall survival; HR, hazard ratio; CI, confidence interval; NA, not available

^a^Adjusted for tumor size, cirrhosis, BCLC stage

In particular, gene expression of *CDK1* and *CDK4* were statistically related to OS in the multivariate Cox regression model of TCGA and GSE14520 cohort. Therefore, *CDK1* and *CDK4* were considered to be the genes associated with the prognosis of HCC patients for further joint-effect analysis. Patients in group D (MST = 2456 days, low *CDK1* and *CDK4* expression) had statistically better prognosis than patients in group A (MST = 899 days, high *CDK1* and *CDK4* expression) in TCGA cohort (Table 5 and Fig. 6J, adjusted HR = 0.491; adjusted *P* = 0.003). Similarly, patients in group d (low *CDK1* and *CDK4* expression) had statistically better prognosis than patients in group a (high *CDK1* and *CDK4* expression) in GSE14520 cohort (Table 5 and Fig. 6K, adjusted HR = 0.431; adjusted *P* = 0.002). The CHCC cohort, used as validation for the joint-effects analysis, showed similar results: patients in group IV (low *CDK1* and *CDK4* expression) had better survival (Additional file 11: Table S2 and Fig. 6L, adjusted HR = 0.287; adjusted *P* = 0.002).

**Table 5:** Joint effects analysis of CDK1 and CDK4 expression in HCC patients

Group	CDK1	CDK4	Patient	OS					
				No. of events	MST	HR (95% CI)	P	Adjusted HR^a^ (95% CI)	Adjusted P^a^
TCGA			(n = 370)		days				
A	High	High	135	56	899	1		1	
B	High	Low	50	21	1490	0.769 (0.465–1.271)	0.305	0.704 (0.408–1.216)	0.209
C	Low	High	50	20	1622	0.760 (0.455–1.269)	0.294	0.857 (0.489–1.502)	0.591
D	Low	Low	135	33	2456	0.430 (0.279–0.662)	< 0.001	0.491 (0.307–0.785)	0.003
GSE14520			(n = 212)		months				
a	High	High	80	37	57.9	1		1	
b	High	Low	2	12	52.7	0.989 (0.515–1.897)	0.972	1.170 (0.606–2.258)	0.641
c	Low	High	26	10	NA	0.756 (0.376–1.522)	0.434	0.566 (0.273–1.173)	0.126
d	Low	Low	80	23	NA	0.480 (0.285–0.808)	0.006	0.431 (0.255–0.729)	0.002

OS, overall survival; MST, median survival time; HR, hazard ratio; CI, confidence interval; NA, not available

^a^Adjusted for tumor size, cirrhosis, BCLC stage in GSE14520 cohort; and adjusted for tumor stage and radical resection in TCGA cohort

**Fig. 7:**
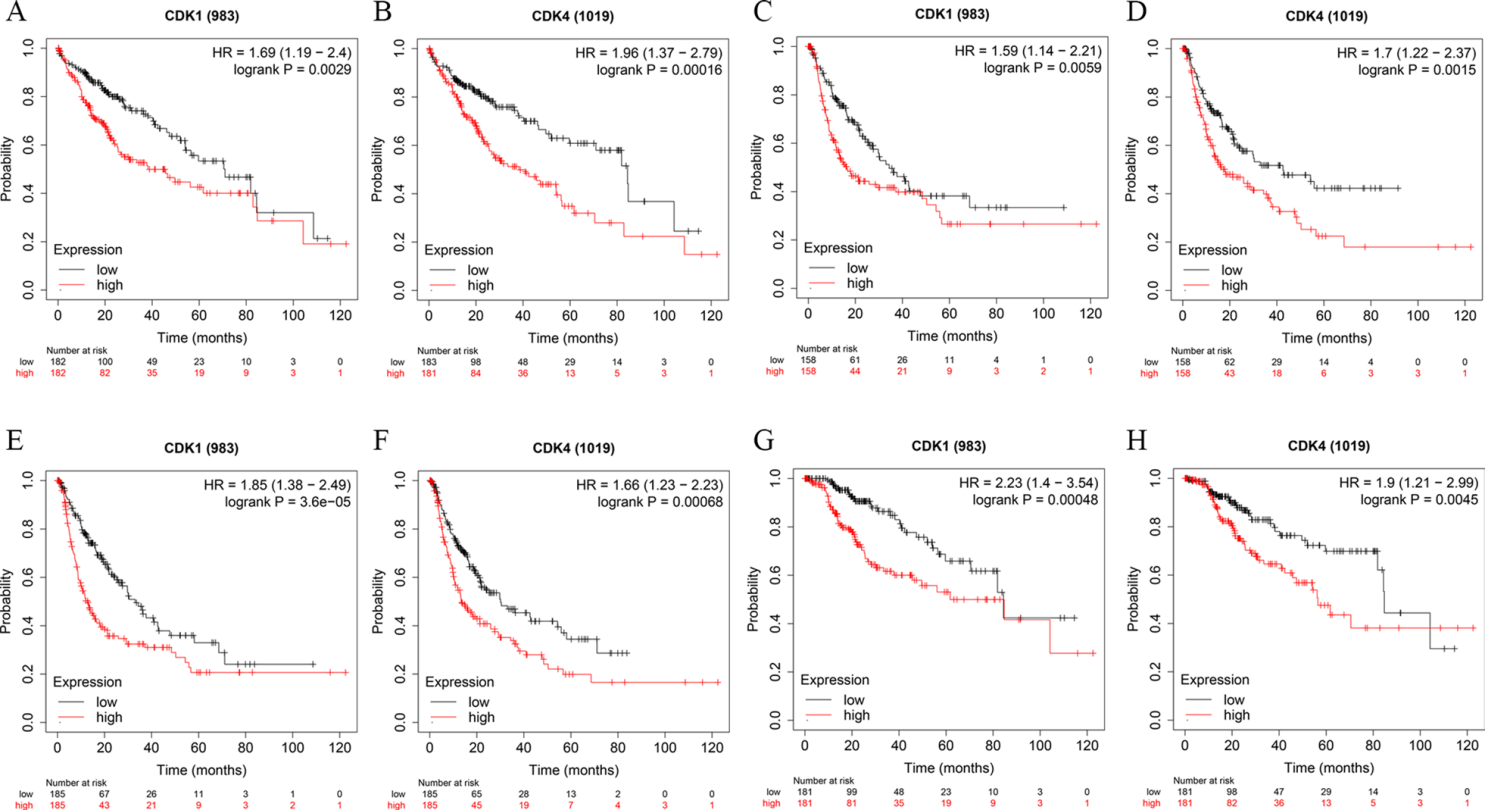
Expression of *CDK1* and *CDK4* in HCC in Kaplan–Meier plotter tool. Overall survival curves were plotted for *CDK1 * (**A**) and *CDK4* (**B**); Relapse free survival curves were plotted for *CDK1* (**C**) and *CDK4* (**D**); Progression free survival curves were plotted for *CDK1* (**E**) and *CDK4* (**F**); Disease free survival curves were plotted for *CDK1* (**G**) and *CDK4* (**H**)

To validate the prognostic value of *CDK1* and *CDK4* genes, we searched the value of *CDK1* and *CDK4* in HCC in multiple datasets. In the CHCC cohort, HCC patients with high expression of *CDK1* (Additional file 3: Figure S3A; *P* < 0.001) and *CDK4* (Additional file 3: Figure S3B; *P* < 0.001) had statistically worse prognosis. In TIMER2.0 results as shown in Additional files 4, 5, 6: Figure S4-6, *CDK1* and *CDK4* showed high expression in HCC and other cancers and were positively correlated with TP53 gene mutation, a common mutation site in HCC. As the results of the GEPIA website shown in Additional file 7: Figure S7, *CDK1* and *CDK4* were highly expressed in HCC than normal liver tissues and they also were highly expressed in other cancers, and expression of *CDK1* and *CDK4* was positively correlated with the stages of HCC. From the Kaplan–Meier Plotter website, we obtained the survival curves of *CDK1* and *CDK4*, and the results showed that HCC patients with high expression of *CDK1* and *CDK4* had shorter OS (Fig. 7A, B), relapse-free survival (Fig. 7C, D), progression-free survival (Fig. 7E, F) and disease-free survival (Fig. 7G, H) than those with low expression, and these results were statistically significant.

### Prognostic signature construction

**Fig. 8:**
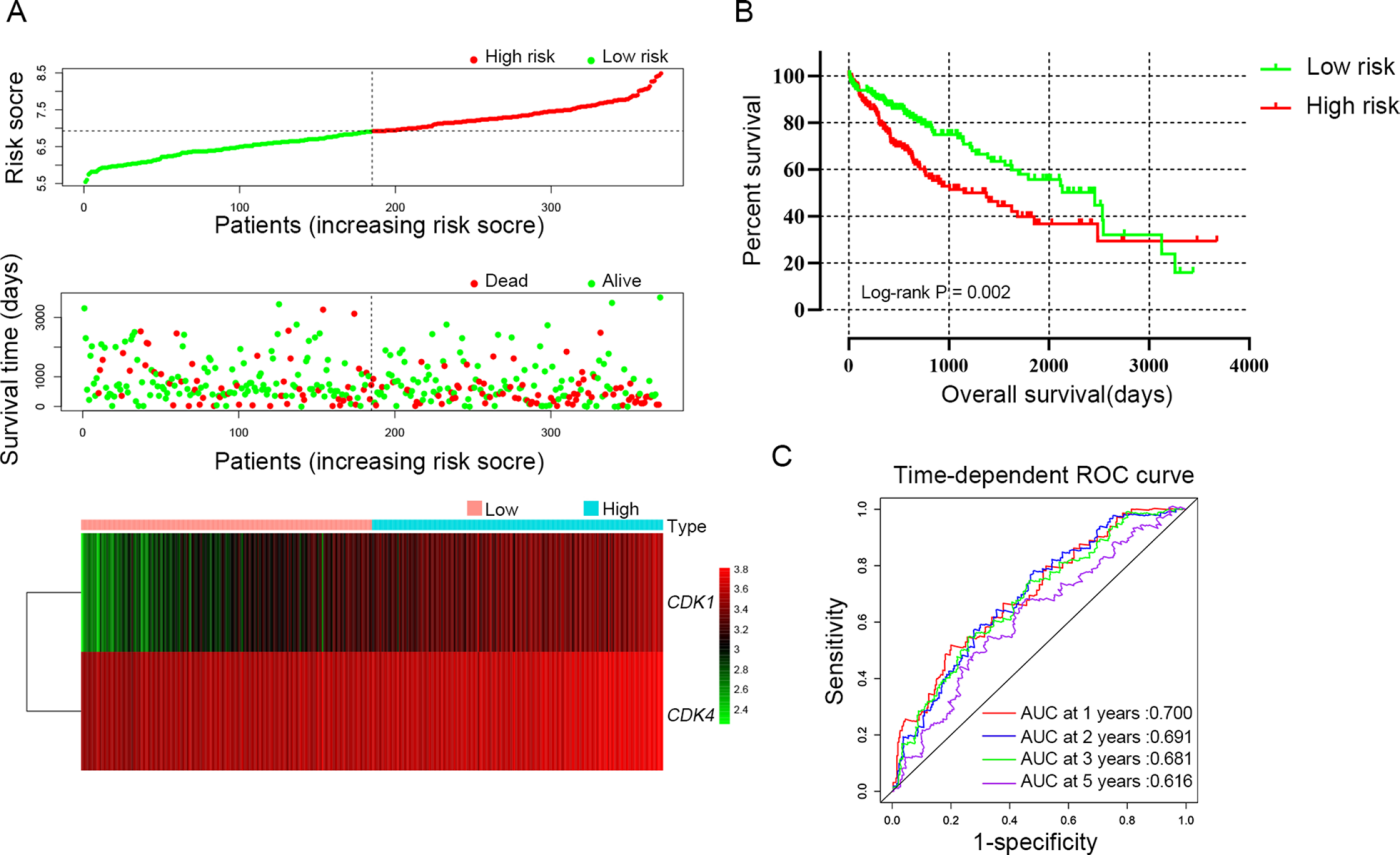
Prognostic risk score models of *CDK1* and *CDK4* genes in HCC patients of TCGA cohort. **A** Risk score from low to high, distribution of patient survival status and risk score and heat map of *CDK1* and *CDK4* genes; **B** Kaplan–Meier survival curves for low-risk and high-risk groups; **C** Time-dependent ROC analysis of the risk score predicts the HCC OS

**Fig. 9:**
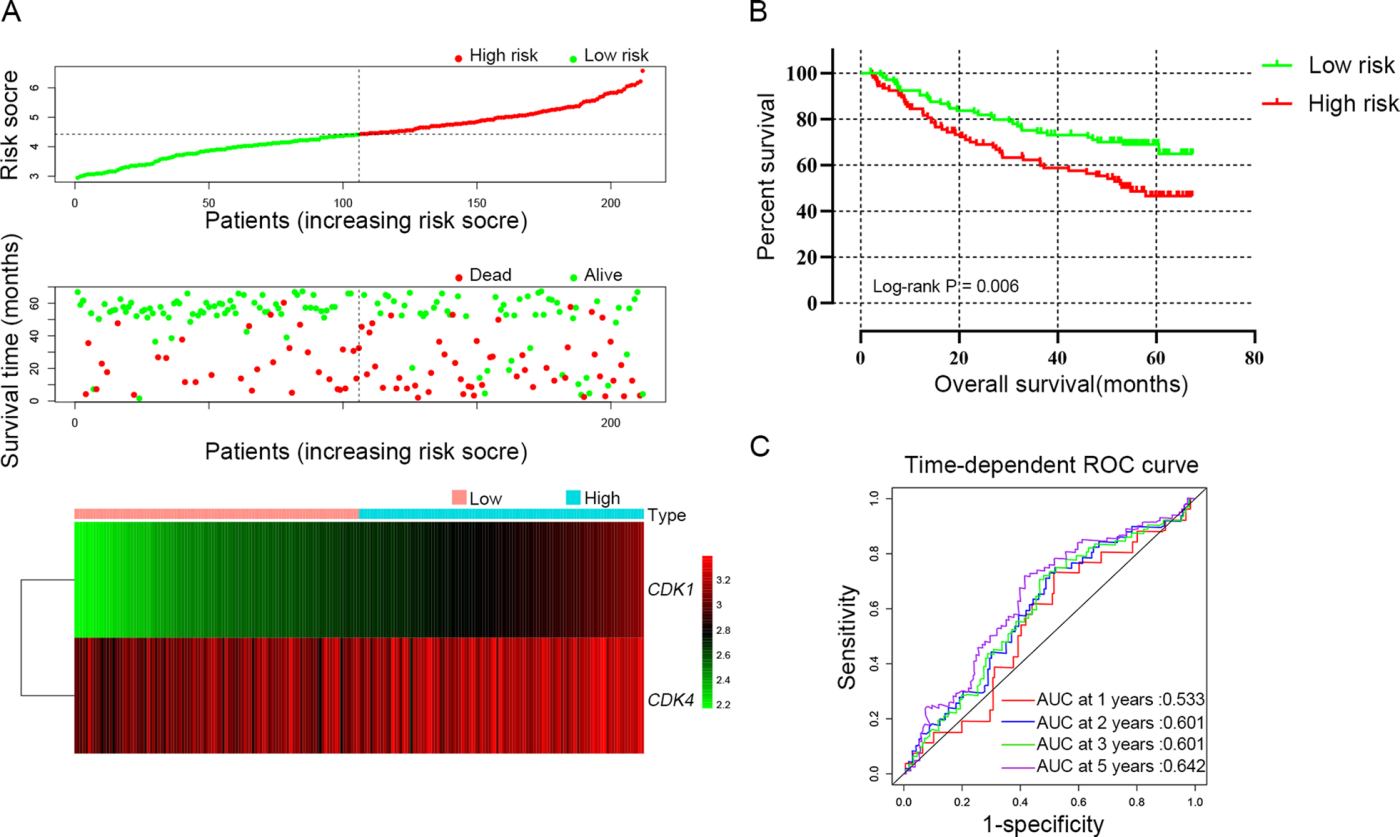
Prognostic risk score models of *CDK1* and *CDK4* genes in HCC patients of GSE14520 cohort. **A** Risk score from low to high, distribution of patient survival status and risk score and heat map of *CDK1* and *CDK4* genes. **B** Kaplan–Meier survival curves for low-risk and high-risk groups. **C** Time-dependent ROC analysis of the risk score predicts the HBV-related HCC OS

Prognostic model was constructed to determine the combined predictive value of *CDK1* and *CDK4* expression. In TCGA cohort, the tumor stage and radical resection were as adjusted factors in the multivariate Cox regression model and regression coefficients (β) of *CDK1* and *CDK4* were calculated. Therefore, risk score = expression of *CDK1* × 0.251 + expression of *CDK4* × 0.444. HCC patients were divided into high-risk group (above the median risk score) and low-risk group (below the median risk score) for the calculation of the relationship between risk score and OS by cox regression model and the results were shown in Table 6 and Fig. 8A, B. Compared with the low-risk group (MST = 2456 days), the high-risk group (MST = 1149 days) showed statistically increased risk of death (adjusted HR = 1.643; adjusted *P* = 0.01) in HCC patients. The model’s predictive value was assessed by time-dependent ROC curves, which had AUC of 0.700, 0.691, 0.681 and 0.616 for the 1-year, 2-year, 3 year and 5-year ROC curves, respectively (Fig. 8C). Similarly, when β was calculated when tumor size, BCLC stage and cirrhosis as adjusted factors, risk score = expression of *CDK1* × 0.792 + expression of *CDK4* × 0.024 in GSE14520 cohort. The results suggested that the high-risk group suffer experience a worse prognosis (adjusted HR = 2.237; adjusted *P* < 0.001 Table 6 and Fig. 9A, B). The AUC was 0.533, 0.601, 0.601 and 0.642 for 1-year, 2-year, 3 year and 5-year ROC curves, respectively (Fig. 9C).

**Table 6:** Survival analysis of risk scores model in HCC patients

Variables	Patients	NO.of event	MST	HR (95% CI)	P	Adjusted HR ^a^ (95% CI)	Adjusted P ^a^
TCGA	n = 370		Days				
Low risk	185	55	2456	1			
High risk	185	75	1149	1.740 (1.227–2.468)	0.02	1.643 (1.124–2.402)	0.01
GSE14520	n = 212		Months				
Low risk	106	33	NA	1		1	
High risk	106	49	54.8	1.848 (1.187–2.876)	0.007	2.237 (1.424–3.514)	< 0.001

MST, median survival time; HR, hazard ratio; CI, confidence interval

^a^Adjusted for tumor size, cirrhosis, BCLC stage in GSE14520 cohort; and adjusted for tumor stage and radical resection in TCGA cohort

### GSEA

In the GSEA analysis, GEO and TCGA datasets were sorted according to expression of *CDK1* and *CDK4*, respectively. In the TCGA cohort, the GSEA results suggested that high expression of *CDK1* and *CDK4* was correlated with cell cycle, liver cancer survival, cell cycle checkpoints, DNA replication and cell cycle G2 and M phase transition (Additional file 8: Figure S8A–L). In GSE14520 cohort, the GSEA results suggested high expression of *CDK1* and *CDK4* was correlated with cell cycle, liver cancer survival, DNA repair, regulation of TP53 activity and viral gene expression (Additional file 9: Figure S9A–L).

### RT-qPCR experiment

**Fig. 10:**
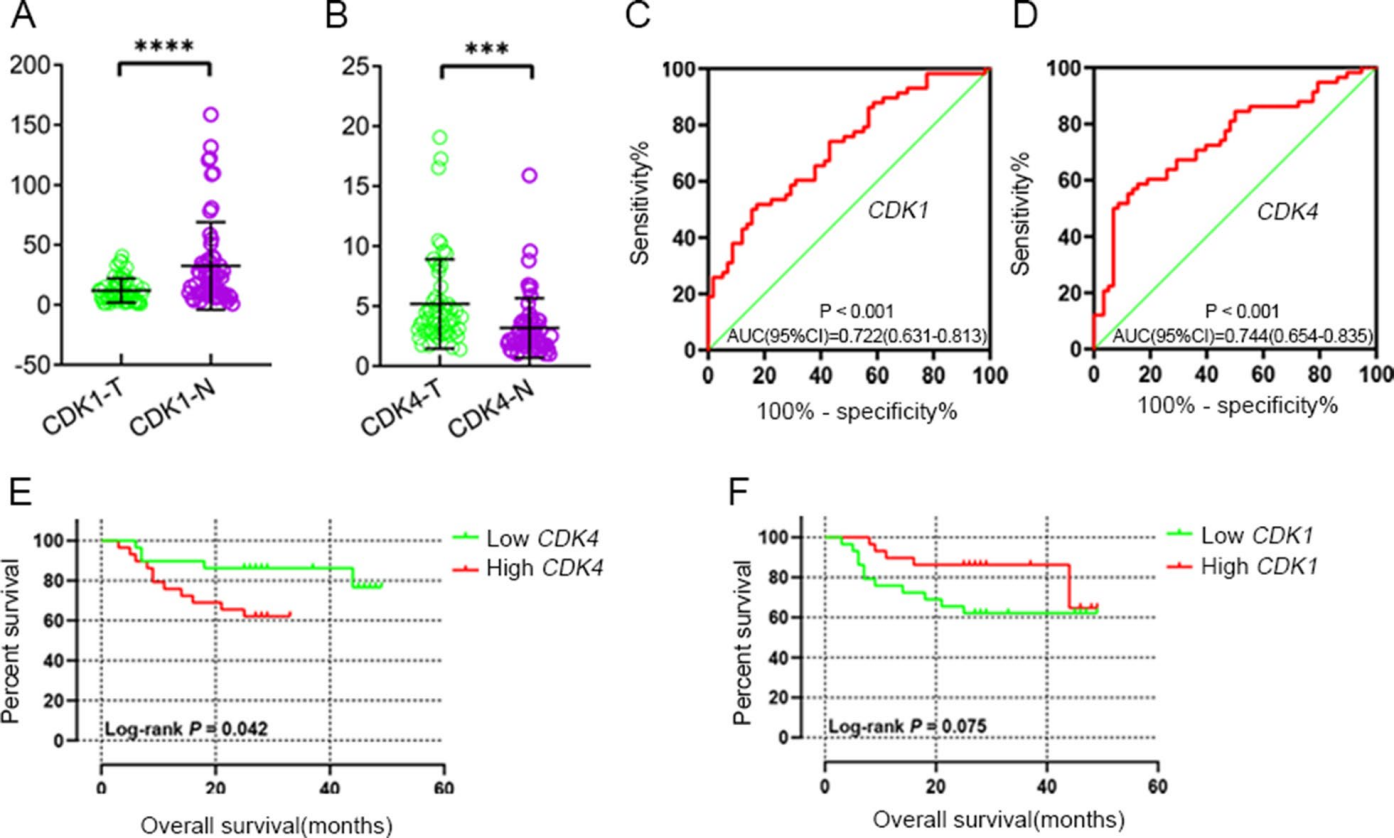
Scatter plot of expression level of *CDK1 *(**A**) *CDK4* (**B**) genes between tumor tissue and adjacent normal liver tissues in validation cohort. The ROC curves of *CDK1* (**C**) and *CDK4* (**D**) genes in distinguish HCC tumor tissue and adjacent normal tissues of validation cohort. Kaplan–Meier survival curves of *CDK4* (**E**) and *CDK1* (**F**) in HCC of validation cohort. *Note*: NS: p > 0.05; *p < 0.05; **p < 0.01; ***p < 0.001; ****p < 0.0001

*CDK1* and *CDK4* were differentially expressed in the RT-qPCR experiment (Fig. 10A, B), respectively (*P* < 0.001), whereas only *CDK4* was overexpressed in HCC tissues consistent with the results from TCGA and GEO cohort. ROC curve analysis suggested that *CDK1* (Fig. 10C, AUC = 0.722, *P* < 0.001) and *CDK4* (Fig. 10D, AUC = 0.744, *P* < 0.001) had statistically predictive value. According to median mRNA expression, patients were divided into high expression group and low expression group. The results of the *CDK4* Kaplan–Meier analysis suggested high expression group had statistically worse prognosis (Fig. 10E, *P* = 0.042), but *CDK1* was not statistically significant (Fig. 10F, *P* = 0.075).

## Discussion

*CDK* gene families were serine/threonine kinases and their main function was involved in cell cycle regulation, which required the specific cyclin subunits to provide domains essential [7]. The results of bioinformatics analysis suggested *CDK1-4, 6* were involved in the regulation of cell cycle and related to the *CCN* gene and protein family, of which *CCNB1*, *CDC20* and *CCND1* were involved in the development of HCC in other reports [38–41]. In addition, KEGG pathway analysis suggested that *CDK1-4, 6* were involved in the p53 signaling pathway and TI3K-Akt signaling pathway.

*CDK* genes mutations often occur in human tumors [9, 42]. It had been reported that *CDK1* interacts with SOX2 to promote tumor initiation in human melanoma and colon [10], and patients with overexpression of *CDK1* were reported to have poor prognosis in epithelial ovarian cancer [43], pancreatic ductal adenocarcinoma [11], lung adenocarcinoma [44] and might be a relevant prognostic biomarker. In addition, *CDK1* was a promising biomarker for metastasis risk in colon cancer [45]. Moreover, lncRNA PVT1 promoted proliferation, migration and invasion of bladder cancer cells by increasing the expression of *CDK1* which down-regulated miR-31 [46]. *CDK4* had been reported to be related to a poor prognosis of osteosarcoma, triple-negative breast cancer, elderly lung cancer, and nasopharyngeal carcinoma [17, 18, 47, 48].

Our study suggested that *CDK1* and *CDK4* were highly expressed in HCC tissues compared to normal controls, and patients with high *CDK1* and *CDK4* expression had poor prognosis. The results of the joint-effects analysis suggested patients with *CDK1* and *CDK4* low expression had better prognosis. In the prognostic model, patients in the high-risk group had worse prognosis. The overexpression of *CDK4* had been verified in the RT-qPCR experiment, but not *CDK1*. Although the clinical significance of *CDK1* was not validated in RT-qPCR experiments, *CDK1* is considered a prognostic factor for HCC in various cohorts and online databases, and its clinical significance in HCC needs to be further explored. Therefore, in this study, HCC patients with overexpression of *CDK4* were considered to have poor prognosis, and *CDK4* might serve as a potential prognostic biomarker of HCC. Previous reports suggested that high levels of *CDK4* can cause hepatic steatosis, fibrosis, and hepatocellular carcinoma in non-alcoholic fatty liver mouse models and patients with fatty liver [49]. *CDK1* had been found highly expressed in HCC tissues, and *CDK1* mediated nuclear accumulation of apoptin and participated apoptosis in cancer [50]. Overexpression of *CDK1* and *CCNB1* can promote HCC cell proliferation and migration through the mitogen-activated protein/extracellular signal-regulated kinase (MEK/ERK) signaling pathway, and trials of MEK1/2 inhibitors for the treatment of HCC are currently underway [51, 52]. JIN et al.'s study suggested that LINC00346 affected p53 signaling pathway by regulating the expression of *CDK1*/*CCNB1* and ultimately regulated apoptosis, invasion and cell cycle of HCC cells [53]. Wu et al. demonstrated that *CDK1* inhibitor RO3306 can increase the antitumor effect of sorafenib in a PDX tumor model, and can provide a basis for personalized treatment for patients with *CDK1*-aberrant HCC [54]. Furthermore, Bollard et al.'s preclinical trials found that Palbociclib, a selective CDK4/6 inhibitor, can promote reversible cell cycle arrest to suppress growth of human liver cancer cell lines [55]. *CDK4* expression had been reported to be associated with histopathologic grade and progression of HCC and can be used as a prognostic marker for HCC [56, 57].

GSEA results of the current study suggested that *CDK1* and *CDK4* are significantly related to liver cancer survival and some mechanisms that might be involved in cancer development: DNA repair, cell cycle, regulation of TP53 activity and viral gene expression. It is well known that the major functions of the *CDK* gene family are involved in cell cycle regulation, and mutations often occur in human tumor cells, of which the most common is *CDK4* [9]. According to previous reports, *CDK4*/*Cyclin D1* can phosphorylate the Ser249 of p53-RS, enhancing the binding of p53-RS and c-Myc, it can thereby activating the c-Myc transcription pathway, and promoting the growth of HCC cells [58]. Studies by Gan et al. showed that CDK1 protein interacts with iASPP protein to affect proliferation and apoptosis of colorectal cancer through p53 pathway [59]. The mechanisms of the CDK1 and p53 pathway in HCC needed further studies.

In our current study, ROC curves suggested that *CDK1* and *CDK4* were sensitive to diagnosis of HCC. At present, α-fetoprotein (AFP) is the serum tumor marker most commonly used for surveillance and early diagnosis of HCC [2]. However, in the retrospective case–control study, even with the most effective cutoff (10–20 ng/mL), the sensitivity was about 60% and the specificity was 80% [2]. Serum AFP > 400 ng/ml was considered to be of diagnostic efficiency, however, the possibility of false-negative results of AFP were high with early-stage HCC [60]. Other serum tumor markers of HCC included des-γ carboxyprothrombin, Golgi protein 73, glypican-3, Neprilysin and AFP-L3, which did not provide better accuracy [2, 60–62]. In recent years, some novel biomarkers of HCC had been discovered, including serum metabolite biomarker panel [63], gut microbiota [64] and serum miRNA (miR-193a-3p, miR-369-5p, miR-672.ect) [65]. In this study, the expression of *CDK1* and *CDK4* in HCC was statistically related to poor prognosis. Therefore, we believe that *CDK1* and *CDK4* might be biomarkers of HCC’s early diagnosis and prognosis prediction.

However, there were some limitations in this study. First, in the RT-qPCR experiment, not all results were consistent with the previous analysis, which resulted from low sample size and other potentially influencing factors. Second, lack of other factors may be involved in the progress of HCC, including smoking status, eating habits, region, drinking status and family history of liver cancer which could be used to further evaluate the relationship between *CDK1-4,6* expression and HCC. Third, this study only explored the relationship between the mRNA expression level of the *CDK* family genes and HCC. Multi-omics analyses of other *CDK* genes such as protein and methylation need to be further explored.

In summary, our study showed that high mRNA expression of *CDK4* was associated with a poor prognosis in HCC patients. *CDK4* may showed as a potential prognostic biomarker of HCC.

## Supplementary Information

**Additional file 1**. **Figure S1:** KEGG pathway map of *CDK1-4, 6* in cell cycle pathway.

**Additional file 2**. **Figure S2:** KEGG pathway map of *CDK1-4, 6* in p53 signaling pathway.

**Additional file 3**. **Figure S3:** Overall survival curves of *CDK1* and *CDK4* in CHCC cohort.

**Additional file 4**. **Figure S4:** *CDK1* gene expression in cancers from the TIMER2.0 website.

**Additional file 5**. **Figure S5:** *CDK4* gene expression in cancers from the TIMER2.0 website.

**Additional file 6**. **Figure S6:** The association of *CDK1* and *CDK4* gene expression with *TP53* gene mutation in cancers from TIMER 2.0 website.

**Additional file 7**. **Figure S7:** Box plot, pathological stage plot and gene expression profile for *CDK1* and *CDK4* from GEPIA website.

**Additional file 8**. **Figure S8:** GSEA results of *CDK1* and *CDK4* in HCC patients of TCGA cohort.

**Additional file 9**. **Figure S9:** GSEA results of *CDK1* and *CDK4* in HCC patients of GSE14520 cohort.

**Additional file 10**. **Table S1:** Clinical data of 159 HCC patients in the CHCC cohort.

**Additional file 11**.Table S2. Joint effects analysis of *CDK1* and *CDK4* expression in CHCC cohort.

## Data Availability

The datasets generated and analysed during the current study are available from University of California, Santa Cruz Xena, https://xenabrowser.net/datapages/; Gene Expression Omnibus, https://www.ncbi.nlm.nih.gov/geo/query/acc.cgi?acc=GSE14520 and The National Omics Data Encyclopedia, https://www.biosino.org/node/project/detail/OEP000321.

## Abbreviations

HCC: Hepatocellular carcinoma
CDK: Cyclin-dependent kinases
RT-qPCR: Revers transcription-quantitative PCR
MST: Median survival time
HR: Hazard ration
TCGA: The Cancer Genome Atlas
GEO: Gene expression omnibus
GO: Gene Ontology
CHCC: Chinese hepatocellular carcinoma
T2DM: Type 2 diabetes
NAFLD: Non-alcoholic fatty liver
KEGG: Kyoto Encyclopedia of Genes and Genomes
PPI: Protein–protein interaction
ROC: Receiver operating characteristic
OS: Overall survival
AUC: Area under the curve
GSEA: Gene set enrichment analysis
CI: Confidence interval
HBV: Hepatitis B virus
β: Regression coefficient
AFP: α-Fetoprotein
