# Supplementary figures and images for: Clinical implications and molecular mechanisms of Cyclin-dependent kinases 4 for patients with hepatocellular carcinoma
Source: BMC Gastroenterol. 2022 Feb 22;22:77. doi: 10.1186/s12876-022-02152-w (PMC8864914; doi:10.1186/s12876-022-02152-w)

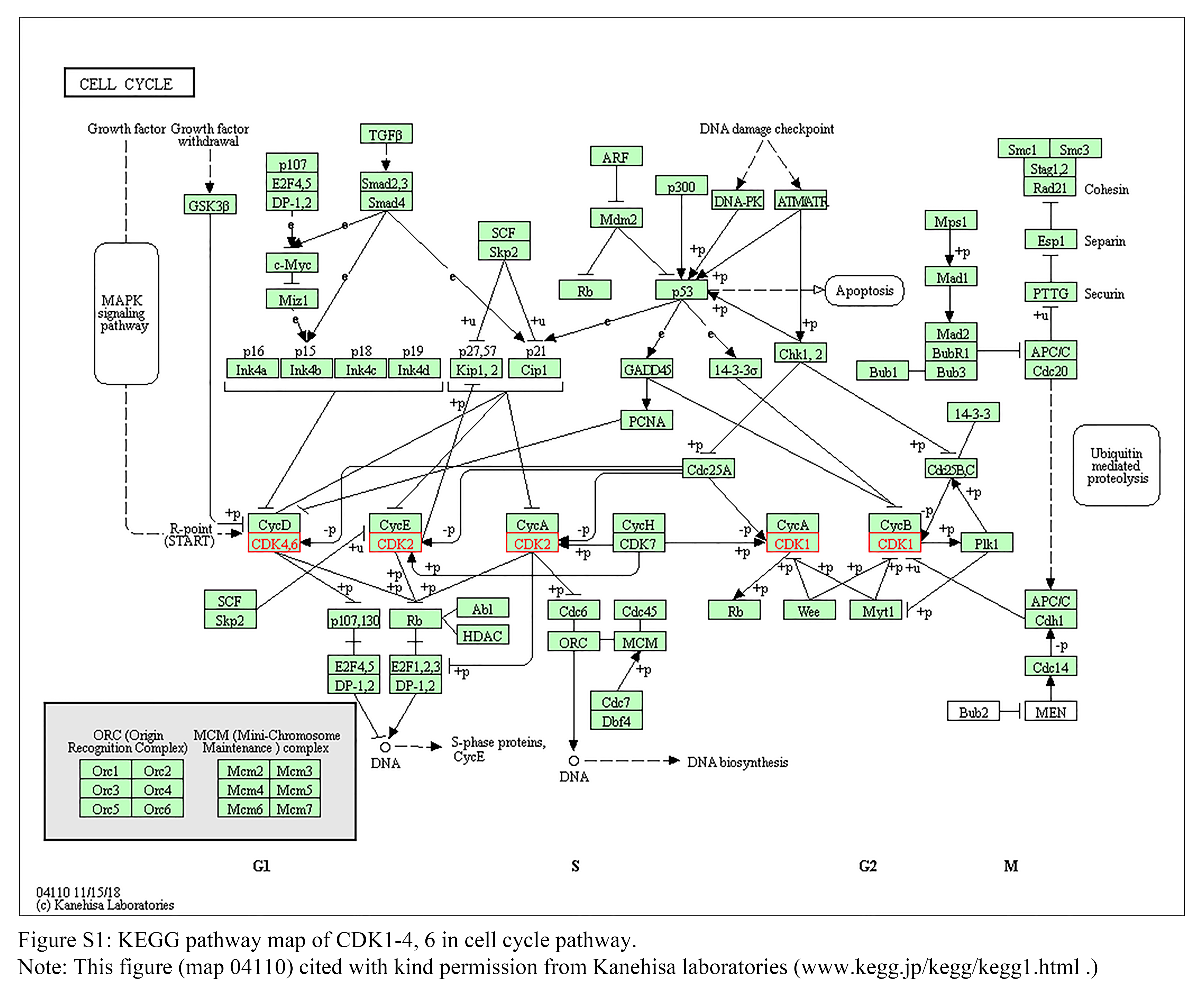

Supplement: Supplementary file 1 — Additional file 1. Figure S1: KEGG pathway map of CDK1-4, 6 in cell cycle pathway. [file 12876_2022_2152_MOESM1_ESM.tif]

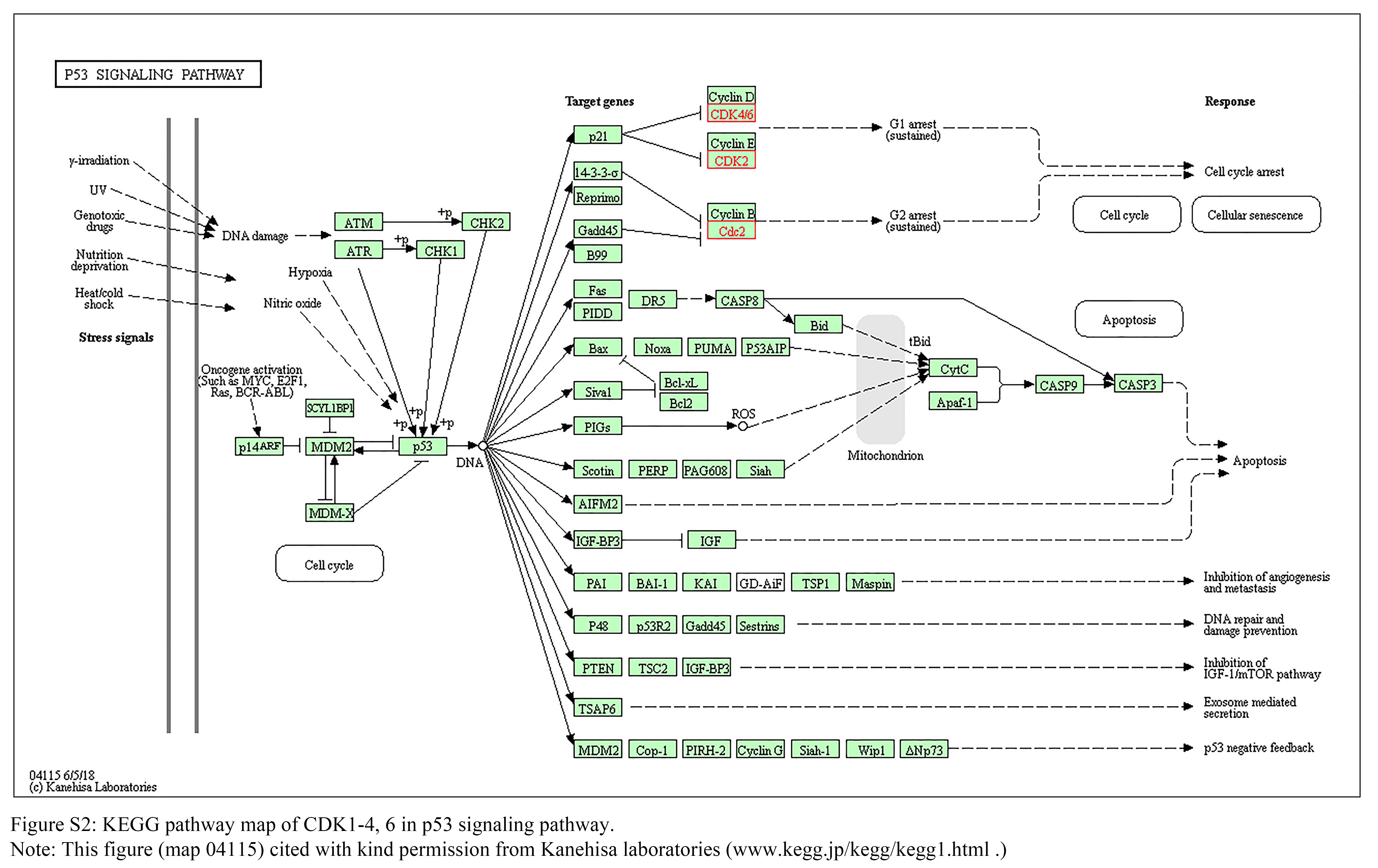

Supplement: Supplementary file 2 — Additional file 2. Figure S2: KEGG pathway map of CDK1-4, 6 in p53 signaling pathway. [file 12876_2022_2152_MOESM2_ESM.tif]

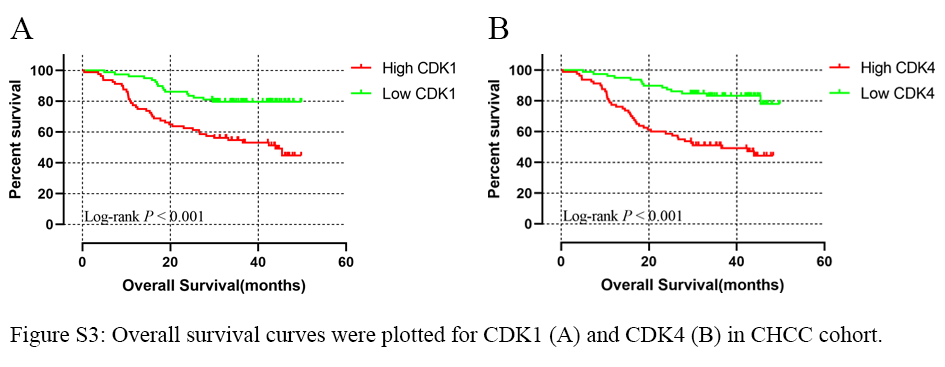

Supplement: Supplementary file 3 — Additional file 3. Figure S3: Overall survival curves of CDK1 and CDK4 in CHCC cohort. [file 12876_2022_2152_MOESM3_ESM.tif]

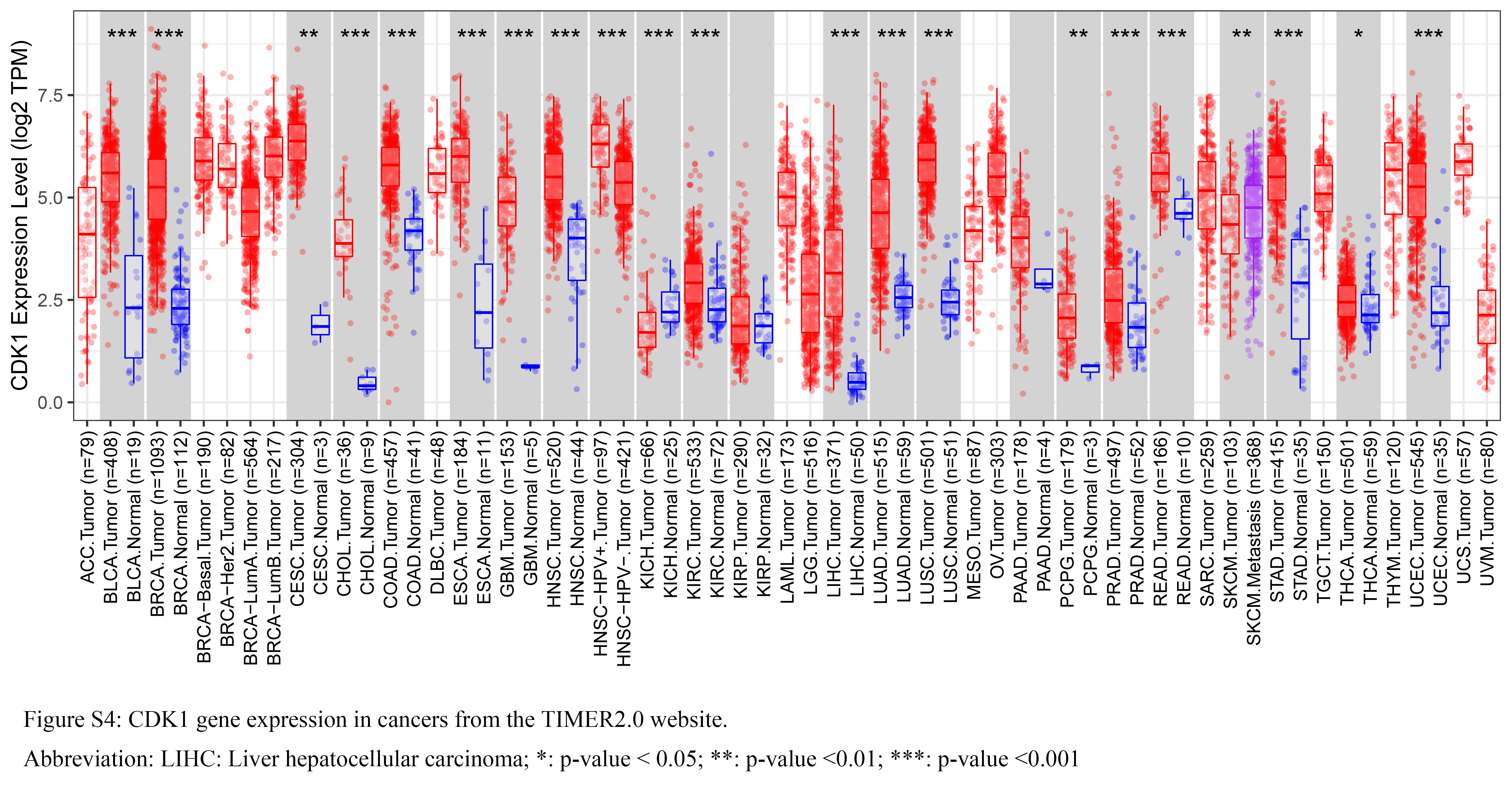

Supplement: Supplementary file 4 — Additional file 4. Figure S4: CDK1 gene expression in cancers from the TIMER2.0 website. [file 12876_2022_2152_MOESM4_ESM.tif]

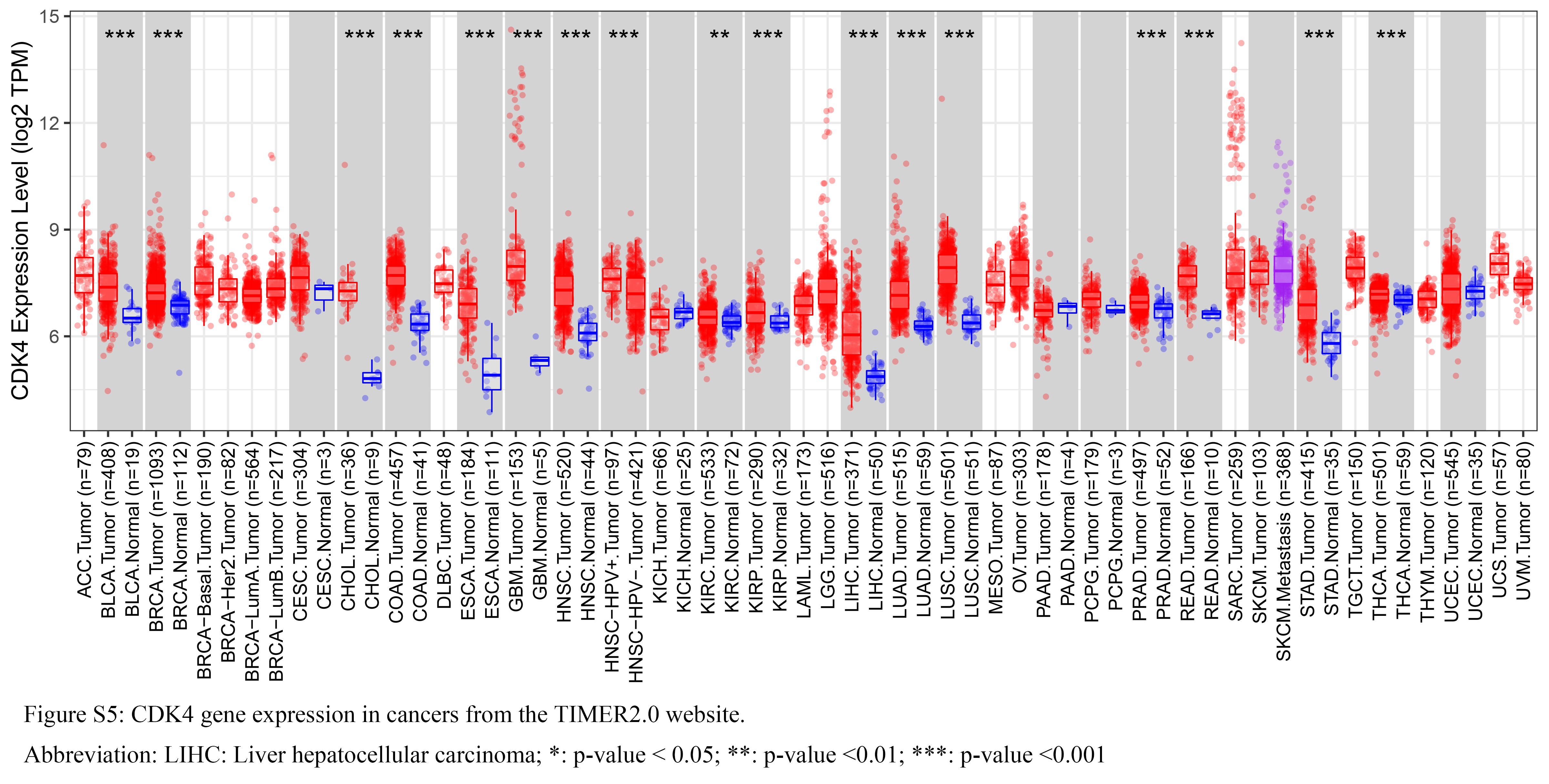

Supplement: Supplementary file 5 — Additional file 5. Figure S5: CDK4 gene expression in cancers from the TIMER2.0 website. [file 12876_2022_2152_MOESM5_ESM.tif]

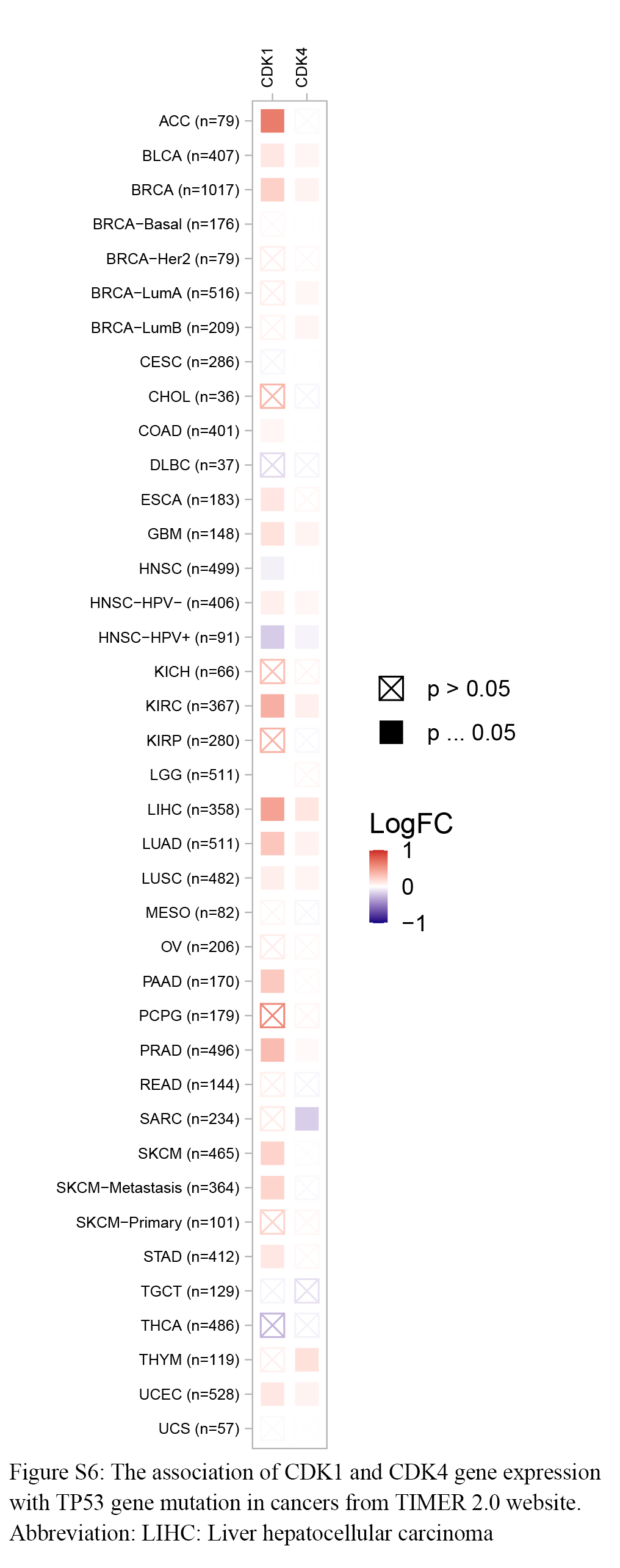

Supplement: Supplementary file 6 — Additional file 6. Figure S6: The association of CDK1 and CDK4 gene expression with TP53 gene mutation in cancers from TIMER 2.0 website. [file 12876_2022_2152_MOESM6_ESM.tif]

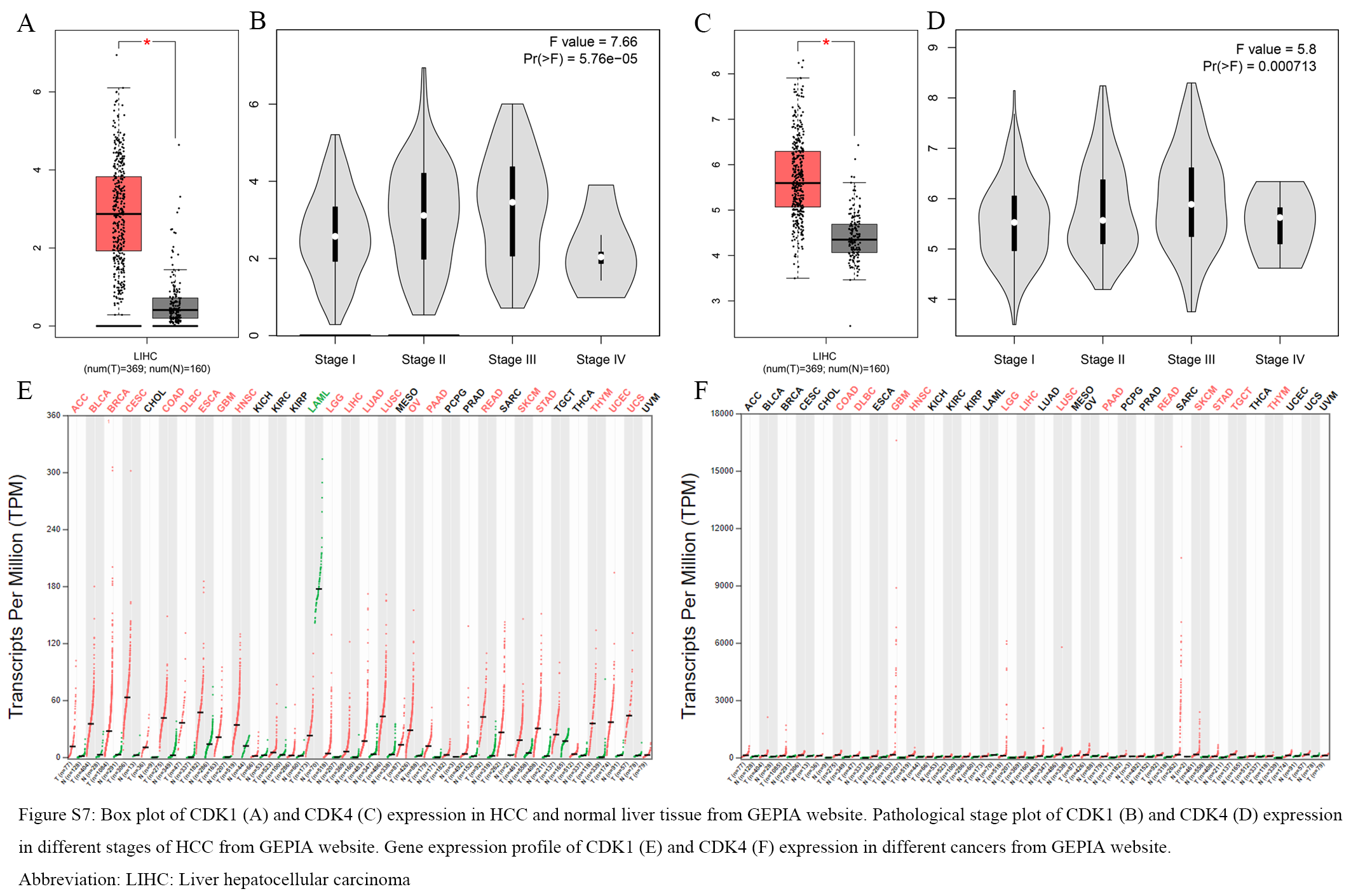

Supplement: Supplementary file 7 — Additional file 7. Figure S7: Box plot, pathological stage plot and gene expression profile for CDK1 and CDK4 from GEPIA website. [file 12876_2022_2152_MOESM7_ESM.tif]

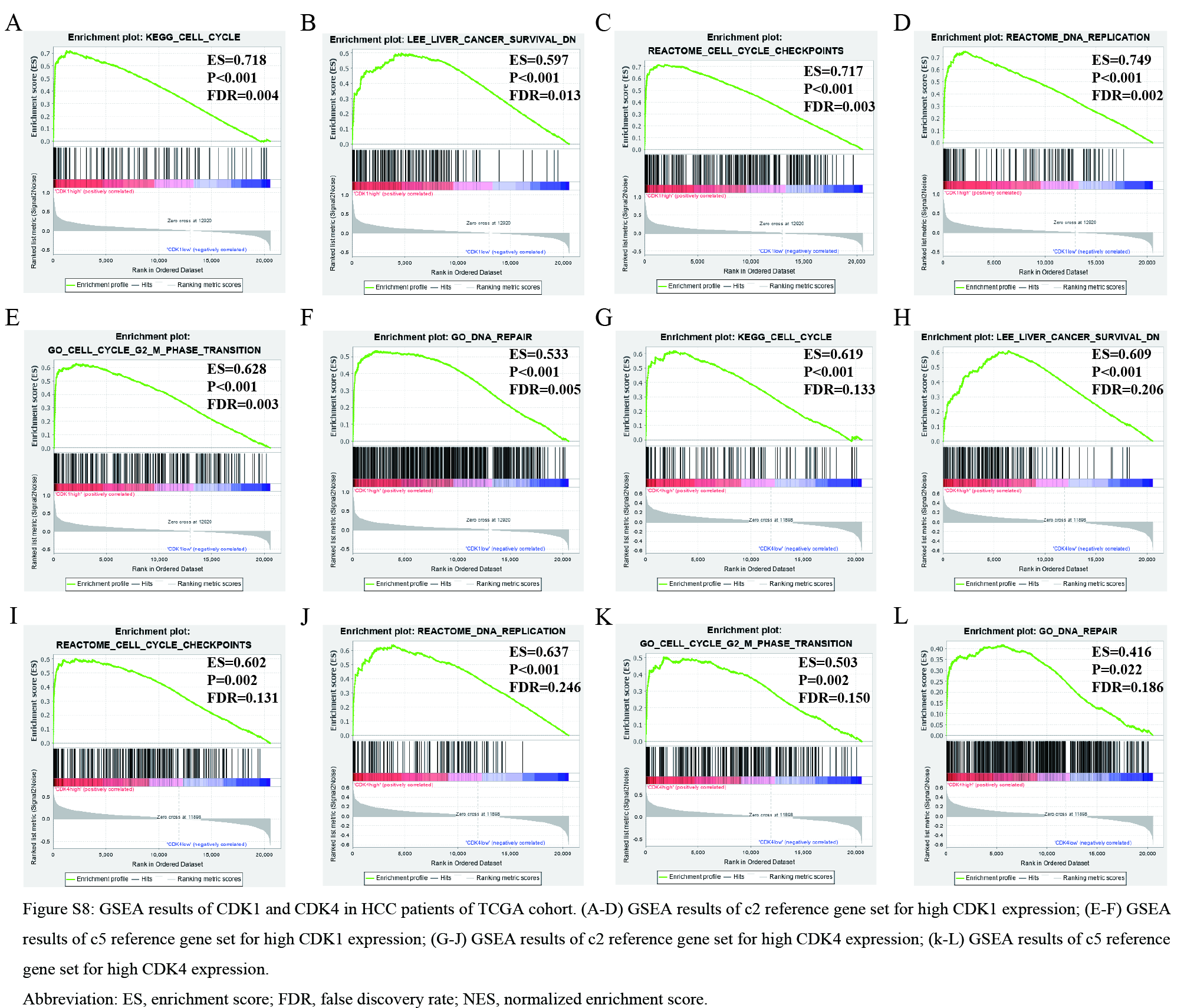

Supplement: Supplementary file 8 — Additional file 8. Figure S8: GSEA results of CDK1 and CDK4 in HCC patients of TCGA cohort. [file 12876_2022_2152_MOESM8_ESM.tif]

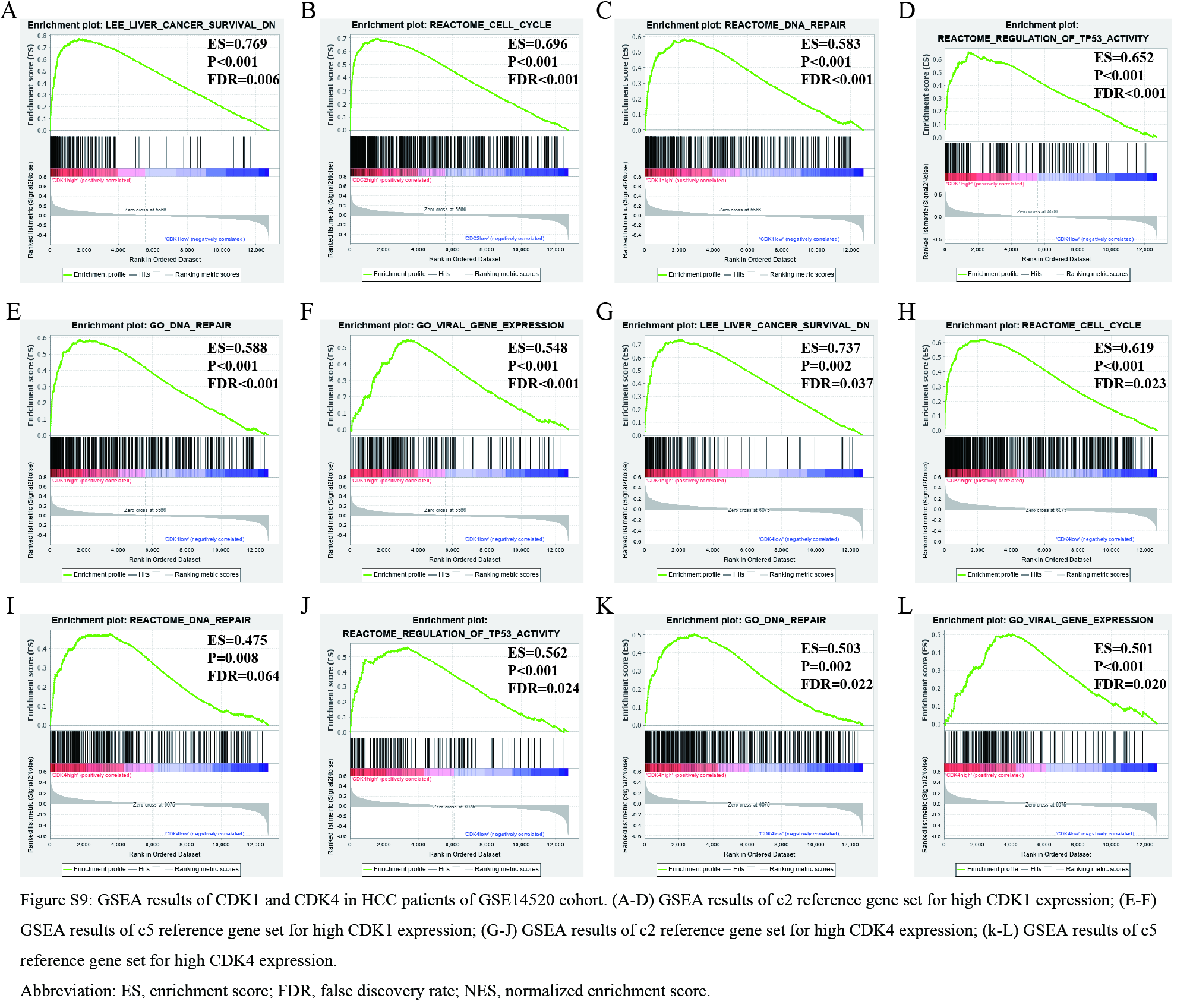

Supplement: Supplementary file 9 — Additional file 9. Figure S9: GSEA results of CDK1 and CDK4 in HCC patients of GSE14520 cohort. [file 12876_2022_2152_MOESM9_ESM.tif]
