# Supplementary material for: Clinical implications and molecular mechanisms of Cyclin-dependent kinases 4 for patients with hepatocellular carcinoma
Source: BMC Gastroenterol. 2022 Feb 22;22:77. doi: 10.1186/s12876-022-02152-w (PMC8864914; doi:10.1186/s12876-022-02152-w)
Supplement: Supplementary file 10 — Additional file 10. Table S1: Clinical data of 159 HCC patients in the CHCC cohort. [file 12876_2022_2152_MOESM10_ESM.docx]

Table S1: Clinical data of 159 HCC patients in the CHCC.

| Variables | patient（n=159） | OS | | | |
| --- | --- | --- | --- | --- | --- |
|  |  | No. of events | MST (months) | HR (95% CI) | P |
| Age(years) |  |  |  |  |  |
| ≤60 | 106 | 41 | NA | 1 |  |
| >60 | 53 | 15 | NA | 0.671(0.371-1.214) | 0.187 |
| Gender |  |  |  |  |  |
| Female | 31 | 13 | 43.9 | 1 |  |
| Male | 128 | 43 | NA | 0.758(0.407-1.412) | 0.382 |
| Liver cirrhosis |  |  |  |  |  |
| No | 47 | 14 | NA | 1 |  |
| Yes | 112 | 42 | NA | 1.282(0.699-2.350) | 0.422 |
| Tumor number |  |  |  |  |  |
| 1 | 117 | 43 | NA | 1 |  |
| 2-3 | 31 | 7 | NA | 0.552(0.248-1.227) | 0.145 |
| ≥4 | 11 | 6 | 27.5 | 1.647(0.700-3.873) | 0.253 |
| Tumor size |  |  |  |  |  |
| ≤5cm | 76 | 16 | NA | 1 |  |
| >5cm | 83 | 40 | 43.9 | 2.825(1.578-5.056) | **<0.001** |
| Tumor thrombus |  |  |  |  |  |
| No | 122 | 36 | NA | 1 |  |
| Yes | 37 | 20 | 33.2 | 2.190(1.262-3.802) | **0.005** |
| Tumor enapsulation |  |  |  |  |  |
| No | 48 | 18 | NA | 1 |  |
| Complete | 111 | 38 | NA | 0.848(0.483-1.489) | 0.567 |
| Total bilirubin |  |  |  |  |  |
| <17.1 mol/L | 140 | 52 | NA | 1 |  |
| ≥17.1 mol/L | 19 | 4 | NA | 0.486(0.176-1.345) | 0.165 |
| Preoperative AFP |  |  |  |  |  |
| ≤400 ng/mL | 101 | 23 | NA | 1 |  |
| >400 ng/mL | 58 | 33 | NA | 3.527(2.065-6.025) | **<0.001** |
| BCLC stage |  |  |  |  |  |
| A | 68 | 14 | NA | 1 |  |
| B | 52 | 21 | NA | 2.347(1.192-4.624) | **0.014** |
| C | 39 | 21 | 33.2 | 3.412(1.723-6.755) | **<0.001** |
| TNM stage |  |  |  |  |  |
| I | 91 | 29 | NA | 1 |  |
| II | 14 | 2 | NA | 0.375(0.089-1.575) | 0.180 |
| III | 52 | 24 | 43.9 | 1.561(0.907-2.687) | 0.108 |
| IV | 2 | 1 | 10.6 | 1.943(0.264-14.298) | 0.514 |

Abbreviation: BCLC, Barcelona Clinic Liver Cancer; AFP, α-fetoprotein; MST, median survival time; OS, overall survival; HR, hazard ratio; CI, confidence interval; TNM, Tumor Node Metastasis; NA, not available.
