# Supplementary material for: Clinical implications and molecular mechanisms of Cyclin-dependent kinases 4 for patients with hepatocellular carcinoma
Source: BMC Gastroenterol. 2022 Feb 22;22:77. doi: 10.1186/s12876-022-02152-w (PMC8864914; doi:10.1186/s12876-022-02152-w)
Supplement: Supplementary file 11 — Additional file 11.Table S2. Joint effects analysis of CDK1 and CDK4 expression in CHCC cohort. [file 12876_2022_2152_MOESM11_ESM.docx]

Table S2. Joint effects analysis of *CDK1* and *CDK4* expression in CHCC.

| Group | *CDK1* | *CDK4* | Patient  (n=159) | OS | | | | | |
| --- | --- | --- | --- | --- | --- | --- | --- | --- | --- |
|  |  |  |  | No. of events | MST  (months) | HR (95% CI) | P | Adjusted HR ^a^ (95% CI) | Adjusted P ^a^ |
| Ⅰ | High | High | 62 | 35 | 28.2 | 1 |  | 1 |  |
| Ⅱ | High | Low | 18 | 5 | NA | 0.337(0.131-0.862) | 0.023 | 0.458(0.172-1.224) | 0.119 |
| Ⅲ | Low | High | 18 | 7 | NA | 0.566(0.251-1.277) | 0.294 | 0.840(0.352-2.002) | 0.693 |
| Ⅳ | Low | Low | 61 | 9 | NA | 0.192(0.092-0.401) | **<0.001** | 0.287(0.131-0.628) | **0.002** |

Notes: ^a^ Adjusted for tumor size, tumor thrombus, preoperative AFP and BCLC stage in CHCC cohort

Abbreviation: OS, overall survival; MST, median survival time; HR, hazard ratio; CI, confidence interval; NA, not available.
